# Vascularized bone organoids: current advances and a biomimetic platform for osteonecrosis of the femoral head

**DOI:** 10.1038/s41413-026-00579-5

**Published:** 2026-08-19

**Authors:** Yihao Tao, Yuxi Wang, Ping Wang, Bo Wang, Xiao Lv, Yong Feng

**Affiliations:** 1 Department of Orthopedics, Union Hospital, Tongji Medical College, Huazhong University of Science and Technology, Wuhan, China; 2 Department of Rehabilitation, Wuhan No. 1 Hospital, Wuhan, China

**Keywords:** Pathogenesis, Endocrine system and metabolic diseases

## Abstract

Osteonecrosis of the femoral head (ONFH) is a devastating ischemic bone disease. Its pathogenesis is multifactorial, including impaired bone remodeling, chronic inflammation, dysregulated lipid metabolism, and bone marrow edema, all intrinsically linked to vascular dysfunction. However, conventional experimental models fail to fully capture the complex pathophysiology of ONFH. Animal models suffer from interspecies differences and are inherently limited in investigating bone marrow edema and femoral head collapse. Therefore, the complex pathogenesis of ONFH remains poorly understood due to the limitations of existing models. Recently, organoids have emerged as a powerful tool for complex and biomimetic disease modeling, with vascularization strategies developed to enhance their structural and functional maturity. The central role of vascular dysfunction in ONFH underscores the value of vascularized bone organoids (VBOs) as a biomimetic model. In this review, we summarize vascularization strategies for bone organoids, including in vitro approaches, in vivo integration, and bioengineering technologies. For ONFH, VBOs provide an advanced experimental model for elucidating disrupted angiogenesis–osteogenesis coupling, inter-tissue crosstalk, and alterations in intraosseous pressure. VBOs also hold significant value for evaluating pro-angiogenic therapies, advancing regenerative medicine, and guiding personalized treatment. Overall, VBOs offer a promising platform for advancing mechanistic insights and therapeutic development in ONFH.

## Introduction

Osteonecrosis of the femoral head (ONFH) is an ischemic bone disorder characterized by bone necrosis due to vascular impairment. ONFH can be broadly classified into traumatic and non-traumatic types, the latter most commonly associated with prolonged glucocorticoid (GC) exposure or excessive alcohol consumption, but also reported in systemic conditions such as sickle cell disease, systemic lupus erythematosus (SLE), graft versus host disease (GVHD), HIV infection, and radionecrosis.^1^ The incidence of ONFH has shown an increasing trend in recent years, and GC exposure is recognized as a major contributing risk factor.^2,3^ Despite partial symptomatic relief achieved by early interventions, these strategies fall short of re-establishing functional vasculature or restoring bone structural integrity, which frequently culminates in femoral head collapse and eventual joint replacement. The primary barrier to effective treatment lies in the limited understanding of the pathogenesis of ONFH.^4,5^ The pathogenesis of ONFH is multifactorial, including impaired bone remodeling, chronic inflammation, dysregulated lipid metabolism, and bone marrow edema, all intrinsically linked to vascular dysfunction. However, conventional experimental models fail to fully capture the complex pathophysiology of ONFH. Animal models are constrained by interspecies differences, such as variations in vascular architecture, biomechanical loading, and hormone receptor expression. They are also inherently limited in investigating bone marrow edema and femoral head collapse. Moreover, cell models fail to recapitulate the complex inter-tissue interactions.^6^ Therefore, the complex pathogenesis of ONFH remains poorly understood due to the limitations of existing models. Accordingly, more physiologically relevant human-based models are required, and organoids have recently emerged as promising platforms for complex and biomimetic disease modeling.^7^

Vascularized organoids, which incorporate functional vascular networks, exhibit enhanced nutrient and oxygen exchange as well as improved metabolic waste removal, thereby achieving greater physiological fidelity and extended viability.^8,9^ Vascularization is particularly essential in bone tissue, as blood vessels not only deliver oxygen and nutrients but also actively regulate bone formation, repair, and remodeling through angiogenesis–osteogenesis coupling.^10^ Recently, diverse vascularization strategies have been developed to enhance the maturation of bone organoids, thereby improving their applicability in disease modeling and bone defect repair.^8,11^

Given that vascular dysfunction represents a central event in ONFH,^12,13^ vascularized bone organoids (VBOs) offer an emerging biomimetic model for the mechanistic investigation of ONFH. Recent studies have employed VBOs to dissect the pathological mechanisms of ONFH and to identify potential therapeutic candidates.^14–16^ However, current VBO-based models recapitulate only selected aspects of ONFH complexity and require further refinement. A more comprehensive representation of ONFH requires consideration of disrupted angiogenesis–osteogenesis coupling, vascular-centered pathological cascades, and alterations in intraosseous pressure. Beyond mechanistic insights, VBOs may have regenerative potential for ONFH by potentially promoting coupled angiogenesis and osteogenesis, optimizing the local microenvironment, enhancing bone stability, and maintaining efficacy.

In this review, we summarize recent advances in vascularization strategies for bone organoids, including in vitro approaches (co-culture, scaffold assistance, and endochondral ossification), in vivo integration (in situ and ectopic vascularization), and bioengineering technologies (organ-on-chip and 3D bioprinting). VBOs provide an emerging experimental framework aimed at reconstructing disrupted angiogenesis–osteogenesis coupling, inter-tissue crosstalk, and alterations in intraosseous pressure. VBOs also hold significant value for evaluating pro-angiogenic therapies, advancing regenerative medicine, and guiding personalized treatment.

## Bone vascular biology

Bone vascular structure, function, and formation are essential for the construction of VBOs. Importantly, impaired vascular integrity and disrupted angiogenesis–osteogenesis coupling represent central pathological features of ONFH, which underlie progressive femoral head collapse.^12,13^ Accordingly, bone vascular biology provides the foundation for understanding VBOs and their application in ONFH.

### Bone vascular network

The bone vascular system is essential for bone development and regeneration. Macroscopically, it forms a hierarchical network of afferent arteries, capillaries, and efferent veins.^17^ In long bones, this network comprises the central nutrient artery, metaphyseal–epiphyseal artery, and periosteal artery.^18^ At the microvascular scale, bone capillaries are divided into H-type and L-type vessels.^19^ H-type vessels, which are more prevalent in the metaphysis and subperiosteal regions, play a critical role in supporting bone regeneration.^20–22^ In contrast, L-type vessels, found within the bone marrow cavity, facilitate nutrient exchange and maintain blood flow within the marrow.^23–25^ These two types of vessels interact closely at the metaphysis, contributing to a continuous vascular network that supports bone remodeling and regeneration.^26,27^ Recent studies have identified R-type vessels, a newly recognized type of post-capillary arterioles, which contribute to bone remodeling by supporting vascularization during the repair process.^28^ Collectively, the structural and functional heterogeneity of bone vasculature underlies its regulatory role in bone remodeling and regeneration.^29^

### Angiogenesis in bone

Bone, one of the most highly vascularized tissues in the body, relies on angiogenesis to supply oxygen, nutrients, and osteogenic and osteoclastic precursors to regions of bone formation.^30^ Beyond their conventional roles, bone and its vasculature act as endocrine organs that reciprocally regulate each other.^31,32^ The coupling between angiogenesis and osteogenesis is orchestrated through dynamic crosstalk between endothelial cells (ECs) and bone-resident cells. Within the bone microenvironment, osteoblasts, osteoclasts, mesenchymal stromal cells (MSCs), macrophages, and immune cells release cytokines and growth factors that modulate EC proliferation, migration, and differentiation, thereby shaping angiogenic activity.^33–35^ Conversely, ECs produce osteogenic signals that regulate osteoblast function and contribute to bone formation and remodeling.^36,37^ The cellular interactions and signaling cascades underlying osteogenesis–angiogenesis coupling have been comprehensively reviewed elsewhere^10^ and will not be detailed here.

## Vascularization strategies for bone organoid

Vascularization is essential for bone homeostasis and skeletal development.^25^ VBOs have emerged as an advanced in vitro system capable of recapitulating key aspects of bone physiology and modeling skeletal diseases.^38^ Given the central role of vascular dysfunction in ONFH,^12,13^ VBOs offer an emerging biomimetic platform for advancing mechanistic insights and therapeutic development in ONFH, enabled by the development of various vascularization strategies.

**Fig. 1:**
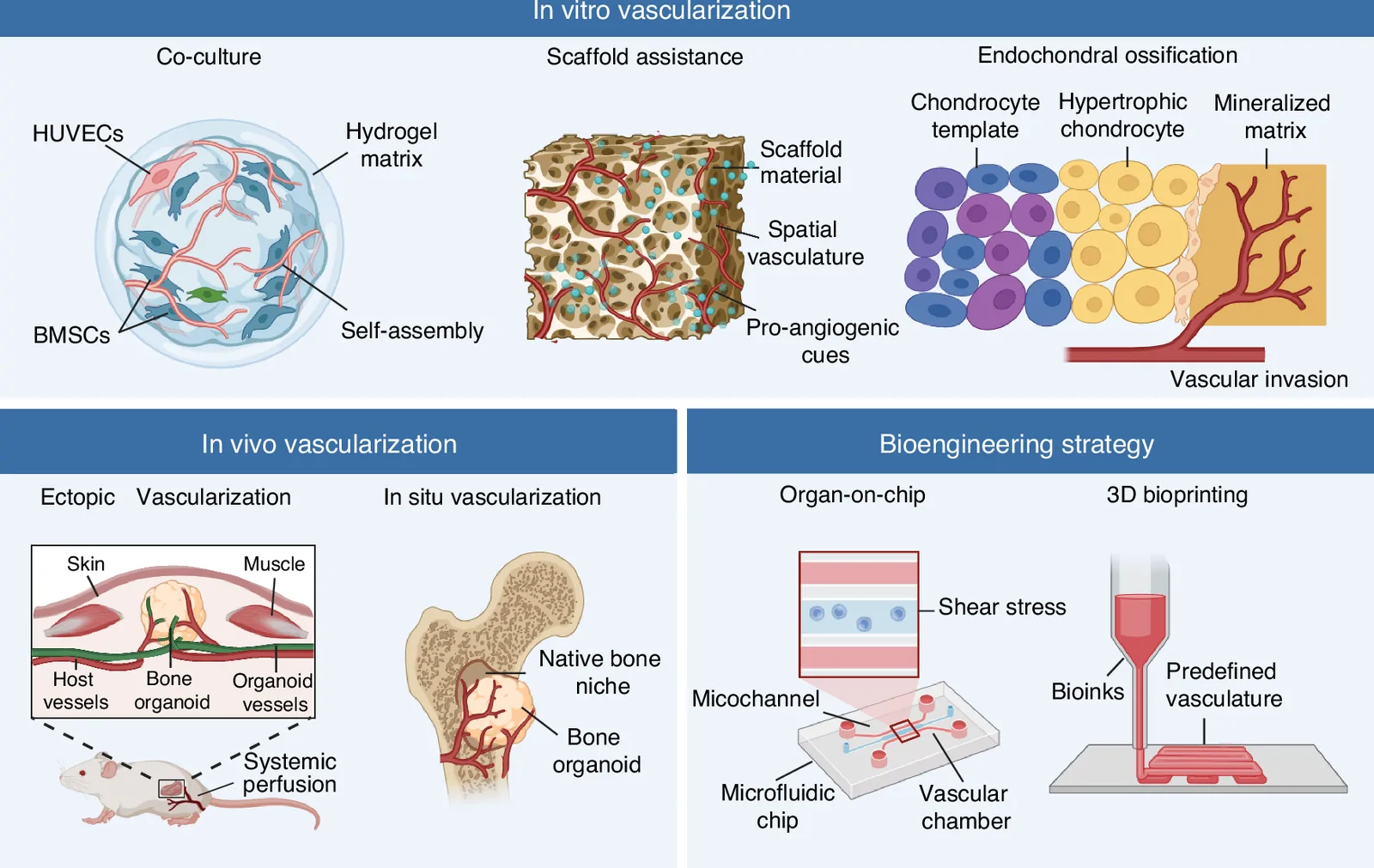
Vascularization strategies for bone organoids. This figure outlines three principal strategies for VBOs: in vitro, in vivo, and bioengineering approaches. In vitro strategies, including co-culture, scaffold assistance, and endochondral ossification, enable the establishment of vascular networks under precisely controlled conditions. In vivo strategies, comprising ectopic and in situ vascularization, utilize host tissues to drive vascular network formation and mature within bone organoids. Bioengineering approaches, involving microfluidic platforms and 3D bioprinting, provide precise spatiotemporal control over vascular patterning

Modeling vascularized bone requires capturing key features of the bone vascular system. Structurally, vascular networks should be spatially integrated within mineralizing bone matrices, forming an organized architecture that supports vascular perfusion. Functionally, angiogenic–osteogenic coupling should reproduce the dynamic interactions between endothelial and bone lineage cells, including vascular invasion during endochondral ossification. At the microenvironmental level, the vascular niche should provide critical cues—such as extracellular matrix (ECM) signals, oxygen tension, ionic regulation, and neural signals—to support vascularization and maintain bone–vascular homeostasis. To recapitulate these key features, researchers have developed a variety of vascularization strategies in bone organoids, including in vitro approaches, in vivo integration, and bioengineering technologies (Fig. 1).

### In vitro vascularization

In vitro vascularization strategies aim to reconstruct vascular networks within bone organoids under precisely controlled conditions. These systems allow precise control over cellular composition and microenvironmental conditions, thereby facilitating the formation of vascular networks. To mimic key structural and functional features of the bone vascular niche, several in vitro vascularization strategies have been developed. These approaches can be broadly categorized into co-culture, scaffold assistance, and endochondral ossification.

#### Co-culture

**Fig. 2:**
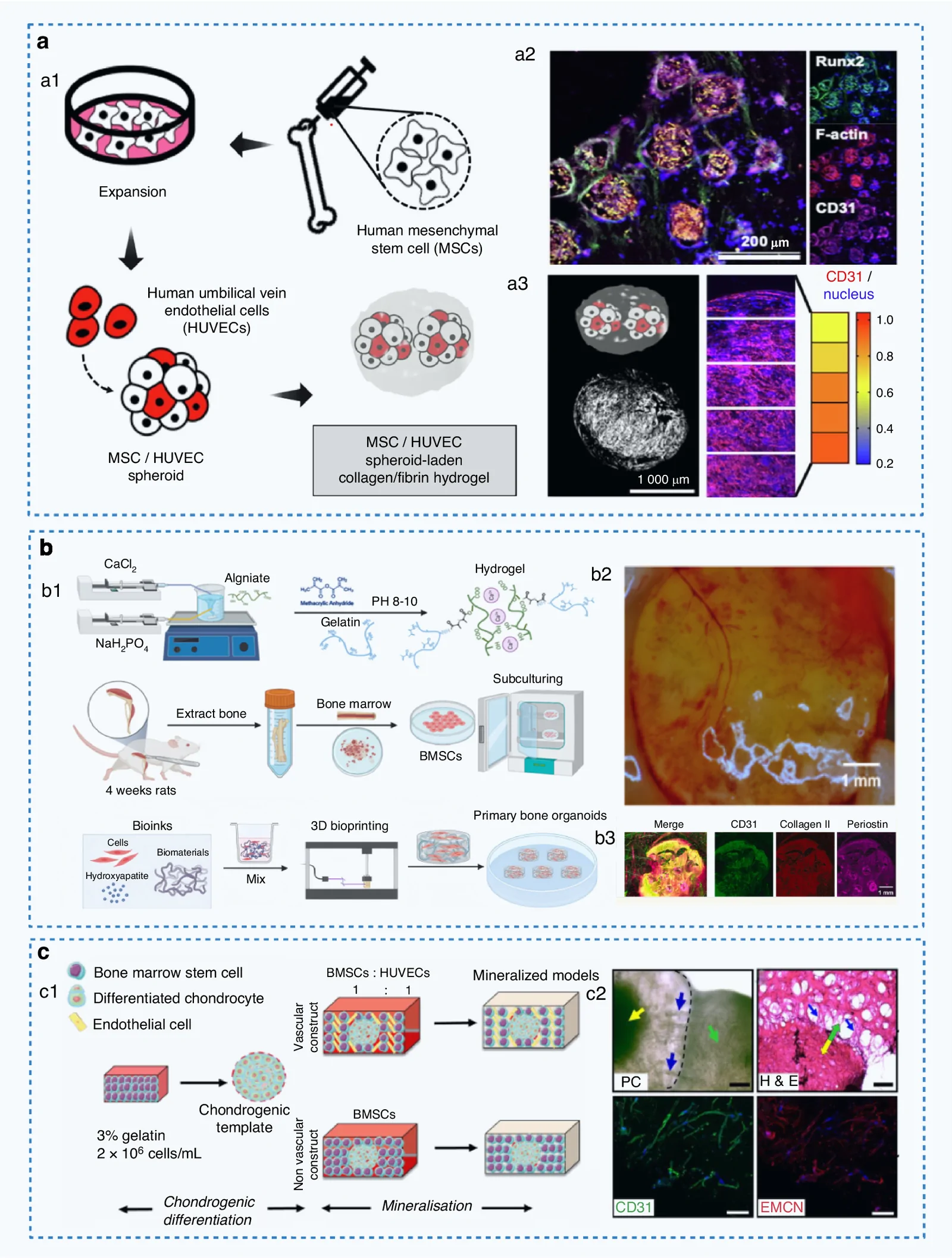
In vitro vascularization strategies for bone organoids. **a** Co-culture approach. (a1) Experimental workflow for generating mesenchymal stromal cell (MSC)/human umbilical vein endothelial cell (HUVEC) spheroids and encapsulation in collagen/fibrin hydrogels. (a2) Immunofluorescence confocal images of MSC/HUVEC spheroids stained for RUNX2, F-actin, and CD31. Scale bar: 200 μm. (a3) 3D reconstructions and corresponding cross-sectional images of MSC/HUVEC spheroids showing CD31 expression. The color scale represents relative fluorescence intensity. Scale bar: 1 000 μm. Reproduced under the terms of the CC-BY Creative Commons Attribution 4.0 International license (https://creativecommons.org/licenses/by/4.0). Copyright 2019, Elsevier.^43^ **b** Scaffold-assisted approach. (b1) Experimental workflow for bioink preparation based on hybrid hydroxyapatite hydrogels. (b2) Stereomicroscopic images of vascularization in bone organoids. Scale bar: 1 mm. (b3) Immunofluorescence images of CD31, collagen II, and periostin in bone organoids. Scale bar: 1 mm. Reproduced from *Bioactive Materials*, Vol. 45, pp. 388–400, 2025, with permission from Elsevier.^63^ **c** Endochondral ossification approach. (c1) Experimental workflow of the endochondral ossification strategy. (c2) Phase-contrast (PC) and H&E staining showing integration of the chondrogenic template (yellow arrow) with the vascularized gelatin layer (green arrow) via multicellular connections (blue arrows). Immunofluorescence images showing CD31 and EMCN expression. Scale bars: 500 μm. Reproduced under the terms of the CC-BY Creative Commons Attribution 4.0 International license (https://creativecommons.org/licenses/by/4.0). Copyright 2025, The American Physiological Society^72^

Co-culture strategies aim to reconstruct intercellular interactions within the bone vascular microenvironment. The fundamental form of co-culture in VBO construction involves the direct integration of endothelial lineages with parenchymal bone cells.^39^ These systems are designed to establish the functional coupling between angiogenesis and osteogenesis, primarily through paracrine signaling-mediated microvascular self-assembly. Human umbilical vein endothelial cells (HUVECs), which are readily accessible and exhibit a strong capacity for tube formation, remain the most widely used EC source. When co-cultured with osteogenic cells, HUVECs accelerate osteogenic differentiation and matrix mineralization (Fig. 2a).^40–45^ Beyond HUVECs, cells with endothelial differentiation potential have also been used to promote vascularization in VBOs. Human dental pulp stem cells, which continuously express VEGFRs and possess endothelial lineage potential,^46^ have been co-cultured with bone marrow mesenchymal stromal cells (BMSCs). This combination enabled the formation of bone organoids containing capillary-like lumens and markedly enhanced mineralization efficiency compared to avascular models.^47^ Collectively, these two-lineage co-culture models establish the foundational vascular–osteogenic paracrine coupling that serves as the basis for more complex co-culture systems.

Building upon these two-lineage systems, more advanced co-culture models incorporate additional lineages to better recapitulate the multicellular vascular niche of bone, where mesenchymal, immune, and osteogenic lineages collectively regulate vascular formation and stabilization. A tri-lineage system has been developed in which osteoblasts, ECs, and macrophages were separately differentiated from induced pluripotent stem cells (iPSCs) and subsequently co-cultured within a GelMA matrix. Macrophage-derived cytokines, including IL-6, VEGF-C, and MCP-1, modulated the inflammatory niche and enhanced angiogenic activity, thereby promoting coordinated immune–vascular–osteogenic development within the bone organoid.^48^ Another study employed a single iPSC source to generate multiple lineages, giving rise to osteolineage, vascular, lymphoid, and myeloid compartments through staged differentiation. Under physioxia conditions, ECs emerged alongside stromal cells, including MSCs and perivascular cells, as well as hematopoietic lineages, and self-organized into lumen-forming vascular networks supported by CD271^+^ perivascular cells. Stromal-derived factors such as ANGPT1/2 and CXCL12 supported vascular stabilization, and hematopoietic populations contributed to the establishment of a bone marrow-like vascular niche.^49^

Unlike direct co-culture of distinct lineages, staged integration of tissue units more closely reflects the developmentally established spatial hierarchy of native vascularized bone. An inter-organoid co-culture strategy was reported, in which both osteoblastic organoids and vascular fate organoids derived from iPSCs were generated and separately integrated within hydroxyapatite scaffolds. Under combined angiogenic and osteogenic cues, ECs self-assembled into fenestrated, capillary-like networks that were spatially organized within the osteogenic matrix, with NG2^+^/α-SMA^+^ perivascular cells enveloping the vascular structures.^50^ This highlights the advantage of inter-organoid co-culture in reconstructing the spatial hierarchy of native vascularized bone.

To overcome the simplicity of models that rely on exogenously added endothelial sources, researchers have used primary cell fractions to better recapitulate the cellular complexity and biochemical milieu of the bone vascular niche. Cell populations derived from digested rib samples demonstrate the capacity to reassemble into vascularized spherical organoids within Matrigel spontaneously.^51^ Independent of exogenous induction, these primary cells maintain multicellular lineages, including osteogenic, osteoclastic, and endothelial components. The researchers used it to model inflammatory disease and test adenosine receptor agonists as a therapeutic agent.^51^ Similarly, VBOs generated from the adipose tissue-derived stromal vascular fraction, which retain MSCs, pericytes, hematopoietic stem cells (HSPCs), endothelial progenitors, and immune cells, have been shown to form capillary-like vascular networks via self-assembly. These models exhibit key features of a native bone vascular niche, including cellular heterogeneity, multicellular matrix reconstruction, spatially organized vascular structures, and immune integration.^52,53^ Derived directly from primary patient tissues, these systems also provide autologous platforms for individualized disease modeling and therapeutic evaluation.

Overall, co-culture approaches enable self-assembled vascularization without predefined structures and allow direct reconstruction of vascular–bone interactions.^8^ However, co-culture alone is insufficient to establish a three-dimensional bone tissue structure. In addition, integrating cells with mismatched biological origins may introduce competition and signaling interference, thereby limiting precise control over the architecture and functionality of the resulting vascular networks.^54^

#### Scaffold assistance

Scaffolds have been widely employed to emulate key features of the ECM, providing structural and biochemical cues that support bone organoid development.^55^ The incorporation of pro-angiogenic materials further establishes a supportive microenvironment for vascular ingrowth, facilitating the efficient formation of three-dimensional vascular networks within bone organoids.^56^

Scaffold materials that improve the local microenvironment represent an important strategy for promoting vascularization. Hypoxia limits angiogenesis during bone regeneration.^57^ To overcome this limitation, oxygen-releasing biomaterials have been developed to alleviate local hypoxia and promote vascularization. Gelatin methacryloyl (GelMA) hydrogels functionalized with hydrogen peroxide–degrading enzymes enable sustained oxygen release and reactive oxygen species scavenging, thereby enhancing endothelial migration and lumen formation.^58^ Similarly, calcium peroxide–hydroxyapatite composites continuously release oxygen, attenuating inflammation by downregulating TNF-α and IL-6 while upregulating IL-10 and TGF-β, thereby improving the local microenvironment and vascularization within bone organoids.^59^ Scaffold materials can also be engineered to modulate the immune microenvironment of the bone vascular network. Graphene oxide particles induced M2 macrophage polarization and promoted the secretion of VEGF and PDGF-BB, thereby enhancing vascular stability. They also activated the PI3K/Akt and focal adhesion pathways, leading to the upregulation of angiogenesis-related genes, such as VEGF.^42^ In addition, Aloe vera has been reported to promote vascularization indirectly through immunomodulation, where sustained M2 macrophage polarization contributes to enhanced vascular infiltration and microvessel formation.^60^

Calcium is a fundamental component of bone mineralization, and Ca^2+^ signaling regulates vasculogenesis by promoting endothelial proliferation and tube formation.^61^ Hydroxyapatite is commonly integrated into composite scaffolds (Fig. 2b), where its degradation into soluble calcium and phosphate mitigates oxidative stress and reduces endothelial apoptosis.^40,41,44,50,59,62–64^ Yang et al. demonstrated that the infiltration of hydroxyapatite into the bone-layer scaffold of a bone–cartilage organoid activated the PI3K-Akt and MAPK signaling pathways, thereby upregulating angiogenic genes and enhancing vascular invasion.^62^ In addition, enzymatically prepared amorphous calcium phosphate has also been incorporated into hydrogels to release calcium and phosphate ions, thereby promoting angiogenesis and osteogenic differentiation.^58^

Notably, decellularized ECM represents tissue-mimetic natural biomaterials that provide endogenous cues for vascular development. Bone-derived ECM (B-dECM) provides natural protein cues that guide vascular development. The combination of B-dECM and calcium phosphate oligomers continuously released Ca^2+^ ions, which stimulated ECs to secrete angiogenic growth factors. Moreover, this composite scaffold modulated epigenetic states by reducing histone acetylation at the promoters of *SIRT6, NOTCH1*, and *NOTCH4*, thereby activating Notch signaling and facilitating angiogenesis.^45^ Similarly, cartilage ECM acts as a native scaffold, providing tissue-specific cues and structural channels that guide CD31^+^ endothelial progenitor cells to align and form organized tubular vascular networks.^53^

Furthermore, scaffolds engineered to release pro-angiogenic factors facilitate the rapid formation of vascular networks. Zhang et al. achieved synergistic angiogenic effects by integrating both rapid and sustained release of multiple growth factors, including VEGF, substance P, BMP-2, and CGRP. These factors promote angiogenesis directly and enhance vascularization indirectly through osteoblast activation.^65^ Lou et al. designed a spatiotemporally graded hydrogel system to achieve sequential angiogenic–osteogenic coupling. The rapidly degradable GelMA layer loaded with a dimethyloxalylglycine stabilized HIF-1α and initiated angiogenesis at the early stage, while the slowly degradable silk fibroin layer containing nano-hydroxyapatite continuously released Ca^2+^ and PO_4_^3-^ ions to sustain osteogenesis at the later stage.^44^

Given the rigid, mineralized, and load-bearing nature of bone tissue, functionalized scaffold assistance has become a widely used approach for promoting vascularization in bone organoids.^66^ Through precise structural design and microenvironmental regulation, these scaffolds effectively drive the formation of three-dimensional functional vascular networks.^67,68^ However, scaffold assistance alone is insufficient to induce complete angiogenesis and is typically integrated with other strategies to enhance vascularization.^69^

Numerous vascularized materials have been developed for bone tissue engineering to repair bone defects.^66^ Among them, materials such as hydroxyapatite^41,62^ and polycaprolactone^40,41^ have been successfully used to construct VBOs, whereas many others remain to be explored for this purpose. Nevertheless, these unexplored materials provide valuable insights and design cues for developing scaffolds for VBOs. Table 1 summarizes representative vascularized materials applied in bone tissue engineering.

**Table 1:** Summary of biomaterials used in tissue engineering and their pro-angiogenic mechanisms

Material type	Common pro-angiogenic mechanisms	Representative materials	Specific mechanisms	References
Calcium phosphates	Degradation releases Ca^2+^ and phosphate ions, upregulating HIF-1α/VEGF signaling and promoting ATP generation for angiogenesis; reduces oxidative stress and endothelial apoptosis; porosity and stress-induced remodeling facilitate vascular invasion.	Hydroxyapatite	Exhibits slow degradation and sustained ion release, providing long-term structural support and mild pro-angiogenic activity.	^221,222^
		β-tricalcium phosphate	Displays rapid degradation and accelerated ion release, stimulating early angiogenesis.	^223,224^
		Biphasic calcium phosphate	Combines HA and β-TCP phases, enabling tunable degradation kinetics and release of bioactive ions.	^225,226^
Natural polymers	Exhibit high biocompatibility; RGD-containing proteins (collagen, fibrin, silk) promote endothelial adhesion and migration via integrin engagement; polysaccharides (chitosan, alginate) modulate immune microenvironments and M2 macrophage polarization, indirectly enhancing angiogenesis.	Collagen	Serves as a vascular basement membrane analog guiding endothelial tubulogenesis.	^227,228^
		Fibrin	Acts as a provisional matrix that sequesters and gradually releases VEGF and FGF, promoting vascular repair.	^228,229^
		Silk protein	Provides vessel-like elasticity and long-term structural stability, conducive to neovascularization.	^230,231^
		Chitosan	Exhibits antibacterial activity and induces M2 macrophage polarization, creating a pro-angiogenic immune niche.	^227,232^
		Alginate	Commonly crosslinks into hydrogels capable of carrying and releasing angiogenic or anti-inflammatory factors.	^233,234^
Synthetic polymers	Possess tunable mechanical and degradation properties; porous or fibrous architectures guide vessel ingrowth; mild acidic degradation of PLA/PLGA induces controlled inflammation, recruiting macrophages and endothelial progenitors.	Polycaprolactone	Commonly forms nanofibrous scaffolds mimicking ECM architecture, supporting endothelial adhesion and migration.	^235,236^
		Polylactic acid	Undergoes acidic degradation, eliciting moderate inflammation and promoting angiogenic cell recruitment.	^229,237^
		Polylactic acid–glycolic acid copolymer	Enables controlled degradation and is commonly used for sustained release of pro-angiogenic or anti-inflammatory factors.	^238,239^

*β-TCP* β-tricalcium phosphate, *FGF* fibroblast growth factor, *HA* hydroxyapatite, *HIF-1α* hypoxia-inducible factor-1 alpha, *PLA* polylactic acid, *PLGA* polylactic acid–glycolic acid copolymer, *RGD* arginine–glycine–aspartic acid, *VEGF* vascular endothelial growth factor

#### Endochondral ossification

Endochondral ossification, as a fundamental process in embryonic bone development,^70^ provides valuable insights for vascularization strategies. During embryonic development, a transient cartilage template undergoes remodeling into bone following vascular invasion, with angiogenesis playing a pivotal role in this transition.^71^ A cartilage template was first induced from BMSCs, upon which another population of BMSCs was co-cultured with HUVECs to recapitulate the vascular invasion during endochondral ossification (Fig. 2c).^72,73^ Similarly, an early co-introduction strategy has been employed, in which human umbilical cord MSCs and HUVECs are combined at the very start of chondrogenic induction, allowing HUVECs to participate throughout subsequent chondrogenic and hypertrophic maturation in a collagen matrix. This early integration enabled vascular invasion during chondrocyte hypertrophy, increased CD31^+^ vascular structures, and enhanced mineralization.^74^ In contrast, delayed endothelial incorporation after osteogenic maturation has also been reported to emulate vascular invasion during endochondral ossification, producing pre-vascularized constructs that formed mature, lumenized vessels after subcutaneous implantation in mice.^75^ Kesharwani et al. employed a microfluidic chip to model this process. They generated sclerotome–HUVEC (SH) organoids by combining human embryonic stem cell-derived sclerotome with HUVECs overexpressing *VEGFA*. The HUVECs migrated outward from the SH organoids and integrated with the vascular network within the chip. Within these organoids, a spatial organization of central SOX9^+^ chondroprogenitor cells and a peripheral COL1^+^ fibrous layer was observed.^76^ Whelan et al. combined chondrocytes at different stages (early, mature, and hypertrophic) with HUVECs, re-establishing the interactions between cartilage templates and blood vessels at each stage. Perfused capillaries and vascular invasion were observed in the early and hypertrophic groups. Although vascular invasion was attenuated in the mature group, the vascular network induced upregulation of SOX2 and OCT4 in chondrocytes, thereby promoting cartilage reprogramming and subsequent osteogenesis.^77^

iPSCs, generated by reprogramming somatic cells to a pluripotent state, can differentiate into virtually any cell type,^78^ making them a powerful tool for modeling endochondral bone development.^79^ Although iPSCs have been widely used to reconstruct endochondral bone,^80–82^ recapitulating the vascular invasion phase remains challenging. A recent study addressed this challenge by aggregating iPSC-derived SOX9^+^ sclerotomal progenitors in high-density micromass culture with BMP2 and SHH activation, promoting spontaneous condensation and the self-organization of early cartilage templates in a scaffold-free manner. After subcutaneous implantation in mice, the templates underwent chondrocyte hypertrophy and progressed into ossification, while triggering robust host vascular invasion. The resulting grafts exhibited highly organized vascularized bone architectures, including EMCN^+^ sinusoidal endothelial networks intertwined with COL1^+^ trabeculae and a well-defined marrow cavity containing hematopoietic cells.^83^ Despite achieving such physiologically relevant vascularization, this iPSC-based endochondral ossification strategy still relies on in vivo transplantation to drive vascular invasion, underscoring the current challenge of reproducing this critical step entirely in vitro.

Endochondral ossification strategy, which mimics the natural process of bone formation, holds promise for comprehensively reconstructing the bone vascular niche in vitro.^76^ However, full reconstitution of the bone homeostasis microenvironment following vascular invasion in vitro has not yet been achieved, particularly regarding marrow formation, hematopoietic niche, and osteoblast–osteoclast balance. Given that ONFH arises from disruption of the bone vascular microenvironment, accurately recapitulating the normal bone vascular niche prior to precipitous pathological events remains a critical objective for future model refinement. Achieving this goal, however, requires precise spatiotemporal control of multiple signaling pathways, posing challenges such as prolonged culture duration and high technical complexity.^72,73^

### In vivo vascularization

In vivo vascularization strategies utilize the host physiological milieu to drive vascular network establishment within bone organoids. Although in vitro approaches can support endothelial self-assembly and microvascular network formation, reproducing the complexity of physiological perfusion remains challenging. In contrast, in vivo implantation enables host-derived vascular invasion and anastomosis with the systemic circulation, thereby facilitating the establishment of mature, perfused, and functionally integrated vascular networks. Nevertheless, this approach introduces host-dependent variability and reduces experimental controllability. Taken together, such functional vascularization still often requires in vivo implantation at present. Many current approaches, therefore, combine in vitro prevascularization with subsequent in vivo implantation to achieve more physiologically relevant vascularization. In vivo strategies can generally be categorized into ectopic and in situ vascularization based on the implantation environment.

#### Ectopic vascularization

**Fig. 3:**
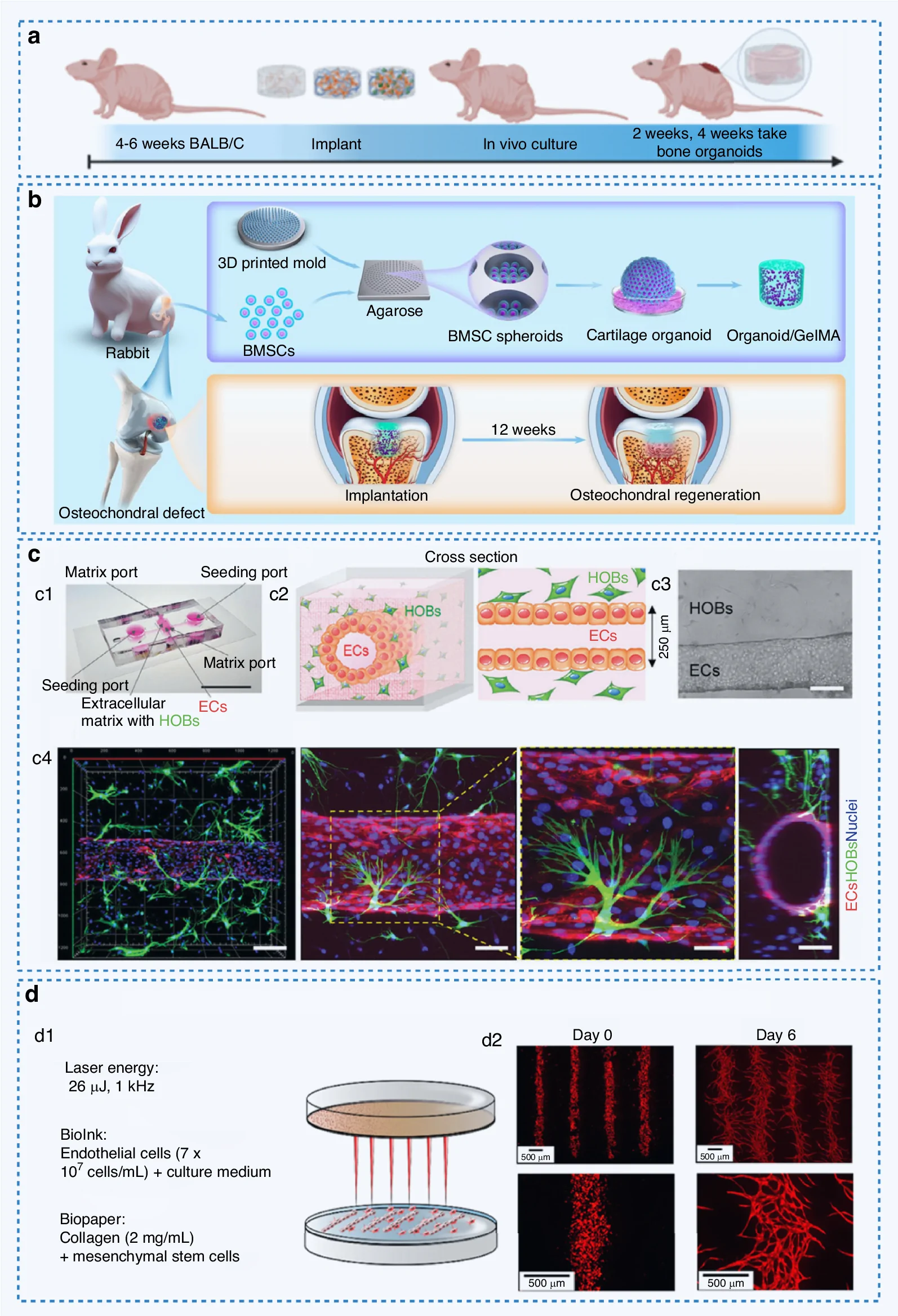
In vivo vascularization and engineering strategies for bone organoids. **a** Ectopic vascularization. Experimental workflow for subcutaneous implantation of bone organoids in mice. Reproduced from *Bioactive Materials*, Vol. 45, pp. 388–400, 2025, with permission from Elsevier.^63^ **b** In situ vascularization. Experimental workflow for cartilage organoid fabrication and implantation into osteochondral defects in rabbits. Reproduced under the terms of the CC-BY Creative Commons Attribution 4.0 International license (https://creativecommons.org/licenses/by/4.0). Copyright 2025, Springer Nature.^89^ **c** Organ-on-chip. (c1) Photograph of 3D microfluidic device using 3D printing scaffold. Scale bar: 1 cm. (c2) Schematic showing the cross-sectional area of the 3D platform. (c3) Representative image of engineered microvessel in a 3D extracellular matrix with human osteoblasts (HOBs) under phase-contrast microscopy. Scale bar: 50 µm. (c4) Representative confocal immunofluorescence images capturing the formed 3D perfusable endothelial vessel (endothelial cells, ECs; red) surrounded by HOBs (green) and nuclei for DAPI (blue). Scale bars: 200 µm (1st picture), 100 µm (2nd and 4th pictures), and 50 µm (3rd picture). Reproduced under the terms of the CC-BY Creative Commons Attribution 4.0 International license (https://creativecommons.org/licenses/by/4.0). Copyright 2025, Wiley.^14^ **d** 3D bioprinting. (d1) Schematic illustration of laser-assisted bioprinting parameters for patterned deposition of ECs. (d2) Fluorescence images showing the formation and maturation of endothelial networks over time. Scale bar: 500 μm. Reproduced under the terms of the CC-BY Creative Commons Attribution 4.0 International license (https://creativecommons.org/licenses/by/4.0). Copyright 2019, Springer Nature^114^

Ectopic vascularization entails implanting bone organoids into highly vascularized, non-osseous tissues, such as subcutaneous tissue or muscle, where the host vasculature drives rapid vascular integration (Fig. 3a).^84^ A primary advantage of this strategy is to establish perfused, functional vascular networks rapidly. Following implantation, endothelial networks within pre-vascularized constructs can quickly form anastomoses with host vessels, allowing red blood cells to enter the lumens of the organoid vasculature and confirming effective perfusion. This host-driven vascular integration supports the formation of mature and functional vascular networks within the organoids.^50,83^

Beyond perfusion, ectopic implantation also facilitates the maturation of bone organoids. The establishment of functional perfusion often promotes bone matrix deposition and the formation of highly mineralized tissue.^58,63,83^ In addition, the ectopic environment has been shown to promote the maturation of bone marrow-like niches, supporting the engraftment, survival, and multi-lineage differentiation of HSPCs.^83^ Furthermore, ectopic implantation has been reported to promote the recruitment of host-derived stromal and supportive cell populations, including MSCs, perivascular cells, and fibroblasts, into VBOs, thereby facilitating vascular maturation and matrix calcification.^65^

A key advantage of ectopic vascularization is implantation within highly vascularized tissues, where abundant host vasculature promotes rapid, robust vascularization during the early post-implantation stage.^85^ In addition, compared with orthotopic implantation into a bone defect, ectopic implantation procedures are technically straightforward and minimally invasive.^40^ However, since implantation occurs outside the native skeletal environment, VBOs are not exposed to bone-specific biomechanical loading, mineral gradients, and biochemical cues, potentially influencing both bone-specific vascular maturation and osteogenic maturation.^86^ Prolonged residence in non-skeletal tissues may also lead to phenotypic drift, as cells within the constructs respond to signals from surrounding adipose and other soft tissues.^87^

#### In situ vascularization

In situ vascularization involves implantation into experimentally created bone defects, where host vasculature supports vascular integration and establishes perfusion within the native bone microenvironment. This strategy leverages the native bone vascular niche to drive coordinated angiogenesis–osteogenesis coupling, thereby promoting the formation of bone-specific vascular networks and organoids that resemble native bone.^88^ Implantation of cartilage organoids into rabbit osteochondral defects enabled the native osteochondral vascular gradient to guide gradient heterogeneous regeneration. This resulted in the formation of an avascular cartilage layer, a calcified cartilage layer, and a vascularized subchondral bone layer. Moreover, CD31^+^/Emcn^+^ H-type vessels were induced within the bone layer. These vessels are recognized as a vascular subtype closely associated with osteogenesis (Fig. 3b).^89,90^ In another study, transplantation of jawbone-like organoids into mandibular defects resulted in the formation of highly organized trabecular structures and complex dendritic osteocyte networks, indicating the establishment of functional bone rather than simple mineralized deposits. In addition, TRAP^+^ osteoclasts were observed within the regenerated bone, suggesting active bone remodeling. Additionally, the newly formed bone exhibited transitional fusion with the host bone and was accompanied by abundant vascularization.^91^

In situ vascularization relies on the native bone niche, where mechanical cues and complex biochemical gradients guide bone-associated angiogenesis and promote the maturation of organoids toward native bone. Moreover, it enables direct structural and functional integration between bone organoids and the bone defect, with potential for bone defect repair.^92,93^ However, compared with ectopic vascularization in highly vascularized tissues, in situ vascularization typically relies on the gradual ingrowth of host vessels, resulting in relatively lower vascularization efficiency.^8^

In the near term, achieving fully mature, perfusable, and functional vascular networks in VBOs will likely continue to rely on integrating in vitro prevascularization with subsequent in vivo implantation. Published studies on in vitro and in vivo vascularization strategies are compiled in Table 2.

**Table 2:** Comprehensive summary of in vitro and in vivo vascularization studies on bone organoids

Vascular methods	Cell sources	Matrix material	Vascular mechanisms	Key metrics	References
Co-culture	SWT	Matrigel	ECs resistant to collagenase digestion directly participating in neovessel formation	CD31^+^vWF^+^	^51^
Co-culture	BMSC DPSC	pNIPAAm	DPSCs consistently expressing VEGFR differentiating toward endothelial lineages	Branch density: 172 mm^−2^ Length density: 17 mm^−1^	^47^
Co-culture	SVF	None	SVF retaining pericytes, hematopoietic stem cells, and endogenous endothelial progenitors, enabling spontaneous vascularization; paracrine and intercellular interactions promoting EC survival and angiogenesis	CD31^+^vWF^+^	^52^
Co-culture	iPSC	GelMA	Co-culture of iPSC-derived ECs, osteoblasts, and macrophages; macrophages secreting IL-6, VEGF-C, MCP-1, and other factors to modulate the inflammatory microenvironment and enhance angiogenesis	VEGFA^+^VEGF-C^+^	^48^
Co-culture	iPSC	Microgel	Same iPSC-derived multi-lineage differentiation generating osteolineage, vascular, lymphoid, and myeloid compartments; physioxia (5% O₂) activating HIF-dependent endothelial differentiation	CD34^+^VEGFA^+^	^49^
Co-culture Scaffold	SVF	CECM	SVF retaining pericytes, hematopoietic stem cells, and endogenous endothelial progenitors, enabling spontaneous vascularization; paracrine and intercellular interactions promoting EC survival and angiogenesis; CECM providing native pro-angiogenic cues	CD31^+^VEGF^+^	^53^
Co-culture Scaffold	BMSC HUVEC	Collagen Fibrin	BMSC–HUVEC co-culture; collagen/fibrin hydrogel enhancing EC spreading, proliferation, and viability; MSC/HUVEC spheroids promoting osteogenesis and coordinated vascular–osteogenic coupling	CD31^+^	^43^
Co-culture Scaffold	BMSC HUVEC	GelMA GO	BMSC–HUVEC co-culture; GO activating PI3K/Akt and focal adhesion signaling, upregulating VEGF and inducing M2 macrophage polarization; M2 macrophages secreting VEGF/PDGF-BB stabilizing nascent vasculature	Branch density: 217 mm^−2^ Node density: 122 mm^−2^ Length density: 5 mm^−1^	^42^
Co-culture Scaffold	BMSC HUVEC	GelMA PCL HAP	BMSC–HUVEC co-culture; HAP degradation releasing Ca^2+^/phosphate, upregulating VEGF/HIF-1α; 200–300 μm pores facilitating capillary-like network formation	CD31+ET1^+^	^41^
Scaffold Ectopic vascularization	BMSC Macrophage	GelMA Laponite nanoclay Aloe vera gel	Plant-derived bioink inducing M2 macrophage polarization, enhancing angiogenesis; synergistic in vivo vascularization	CD31^+^	^60^
Scaffold Ectopic vascularization	BMSC	GelMA-Catalase Y-shaped DNA Ca₃(PO₄)₂	Catalase–GelMA generating O₂ and scavenging ROS; matrix upregulating pro-angiogenic genes (e.g., *COL3A1*); synergistic in vivo vascularization	Branch density: 182 mm^−2^ Node density: 124 mm^−2^ Length density: 5 mm^−1^	^58^
Scaffold Ectopic vascularization	UCMSC	HAP HA	The bone layer activating PI3K-Akt and MAPK pathways, upregulating pro-angiogenic genes; synergistic in vivo vascularization	None	^62^
Scaffold Ectopic vascularization	BMSC	GelMA HA–NB	Outer GelMA shell rapidly releasing VEGF/SP; inner GelMA/HA–NB core providing sustained release of BMP-2/CGRP; synergistic in vivo vascularization	CD31^+^VEGF^+^	^65^
Scaffold Ectopic vascularization	BMSC	GelMA Collagen HAP	Collagen serving as a basement membrane analog guiding endothelial tubulogenesis; HAP degradation releasing Ca^2+^/phosphate, upregulating VEGF/HIF-1α; synergistic in vivo vascularization	CD31^+^VEGF^+^	^63^
Scaffold Ectopic vascularization	BMSC	GelMA AlgMA HAP	HAP degradation releasing Ca^2+^/phosphate, upregulating VEGF/HIF-1α; synergistic in vivo vascularization	CD31^+^ VEGF^+^α-SMA^+^	^64^
Co-culture Scaffold Ectopic vascularization	BMSC HUVEC	Salmon DNA B-ECM CPO	BMSC–HUVEC co-culture; B-ECM/CPO releasing Ca^2+^, upregulating angiogenic growth factors; BECM–DNA–CPO reducing histone acetylation at *SIRT6/NOTCH1/NOTCH4* promoters, activating Notch-driven angiogenesis; synergistic in vivo vascularization	Migration ratio: 80% Node density: 182 mm^−2^ Connection density: 52 mm^2^	^45^
Co-culture Scaffold Ectopic vascularization	ADMSC HUVEC	GelMA PCL HAP	ADMSC–HUVEC co-culture; HAP degradation releasing Ca^2+^/phosphate, upregulating VEGF/HIF-1α; synergistic in vivo vascularization	Vessel density: 155 mm^−2^ Vessel coverage: 70%	^40^
Co-culture Scaffold Ectopic vascularization	BMSC Macrophage	GelMA Silk fibroin HAP-CaO₂	HAP–CaO₂ alleviating hypoxia and reprogramming macrophages toward the M2 phenotype; M2 macrophages improving immune microenvironment; synergistic in vivo vascularization	CD31^+^VEGF^+^	^59^
Co-culture Scaffold Ectopic vascularization	BMSC HUVEC	GelMA Silk fibroin HAP-DMOG	BMSC–HUVEC co-culture; sequential angiogenic–osteogenic coupling via a spatiotemporally graded hydrogel; HAP–DMOG promoting early angiogenesis through HIF-1α/VEGF; synergistic in vivo vascularization	CD31^+^ VEGF^+^ HIF-1α	^44^
Co-culture Scaffold Ectopic vascularization	iPSC	HAP	Co-culture of vascular fate organoids with osteoblastic organoids formed within HAP scaffolds; exogenous supplementation of angiogenic cues (VEGFA), osteogenic signals (Dex, β-GP, and AA2P), IL33, and FGF2; HAP degradation releasing Ca²^+^/phosphate, upregulating VEGF/HIF-1α	Vessel coverage: 35% Vessel diameter: 5–30 μm	^50^
Endochondral ossification	hESC HUVEC	Fibrin Collagen	hESC-derived osteochondral cells connected with the peripheral vascular network in a microfluidic chip, recapitulating early vessel–mesenchymal interactions during endochondral ossification	Vessel density: 148 mm^−2^ Length density: 5 mm^−1^	^76^
Endochondral ossification	BMSC HUVEC	Gelatin	BMSC–HUVEC co-culture on BMSC-derived cartilage template, mimicking early fetal endochondral vascularization	CD31^+^VEGF^+^EMCN^+^	^72^
Endochondral ossification	BMSC HUVEC	Fibrin gel	Microfluidic co-culture of HUVECs with bone organoids at distinct cartilage maturation stages, showing selective vascular invasion into hypertrophic cartilage and upregulating pluripotency factors (SOX2, OCT4)	Vessel coverage: 95% Branch density: 180 mm^−2^ Node density: 120 mm^−2^ Vessel diameter: 105 μm	^77^
Endochondral ossification	UCMSC HUVEC	Collagen	Early UCMSC–HUVEC interactions at the onset of chondrogenic induction, followed by stepwise chondrogenic and hypertrophic maturation; MSC-derived pro-angiogenic cues promoting neovessel formation	Length density: 7 mm^−1^ Branch density: 182 mm^−2^	^74^
Endochondral ossification	BMSC HUVEC	None	BMSC–HUVEC co-culture on BMSC-derived cartilage template, mimicking early fetal endochondral vascularization	CD31^+^ VEGF^+^	^240^
Endochondral ossification Scaffold	BMSC HUVEC	BMSC ECM Gelatin Fibrin	Delayed HUVEC introduction after cartilage template formation and ossification; BMSC ECM and fibrin providing a native pro-angiogenic microenvironment	Node density: 159 mm^−2^ Length density: 13.4 mm^−1^ Branch density: 85 mm^−2^	^75^
Endochondral ossification Ectopic vascularization	BMSC HUVEC	Alginate, calcium sulfate, PCL	BMSC–HUVEC co-culture on BMSC-derived cartilage template, mimicking early fetal endochondral vascularization; synergistic in vivo vascularization	Vessel diameter: 70 μm Vessel volume: 0.5 mm^3^	^241^
Endochondral ossification Ectopic vascularization	iPSC	None	Early cartilage template (iPSC-derived SOX9^+^ sclerotomal progenitors), inducing host vascular invasion in vivo; synergistic in vivo vascularization	EMCN^+^CD31^+^VEGFA^+^	^83^
In situ vascularization	ACPCs BMSC	GelMA AlgMA	Native vascular gradient–guided regeneration with avascular cartilage above and vascularized bone below	CD31^+^EMCN^+^	^90^
In situ vascularization	BMSC	GelMA	Native vascular gradient–guided regeneration with avascular cartilage above and vascularized bone below	CD31^+^vWF^+^	^89^
In situ vascularization	iPSC	None	Transplantation into mandibular bone defects inducing host vascular invasion and vascularized bone maturation	CD31^+^	^91^

Quantitative vascular parameters (e.g., branch density, node density, length density, vessel density) were derived either directly from the cited studies or estimated from published images using ImageJ (NIH, USA). The estimated data are presented for reference and may not exactly reflect the quantitative values reported in the original publications

*AA2P* ascorbic acid-2-phosphate, *ACPS* articular cartilage progenitor cells, *α-SMA* alpha-smooth muscle actin, *ADMSC* adipose-derived mesenchymal stromal cell, *AlgMA* alginate methacrylate, *ALP* alkaline phosphatase, *B-ECM* bone-derived extracellular matrix, *β-GP* β-glycerophosphate, *BMSC* bone marrow mesenchymal stromal cell, *CECM* cartilage extracellular matrix, *CGRP* calcitonin gene-related peptide, *COL1* collagen type I, *CPO* calcium phosphate oligomers, *Dex* Dexamethasone, *DMOG* dimethyloxalylglycine, *DPSC* dental pulp stem cell, *ECs* endothelial cells, *ET1* endothelin-1, *FGF2* fibroblast growth factor 2, *GelMA* gelatin methacryloyl, *GO* graphene oxide, *HAP* hydroxyapatite, *HA–NB* hyaluronic acid–nitrophenyl butanoate conjugate, *hESC* human embryonic stem cell, *iPSC* induced pluripotent stem cell, *UCMSC* human umbilical cord mesenchymal stromal cell, *HUVEC* human umbilical vein endothelial cell, *IL33* interleukin-33, *OCN* osteocalcin, *pNIPAAm* poly(N-isopropylacrylamide), *PCL* polycaprolactone, *ROS* reactive oxygen species, *RUNX2* runt-related transcription factor 2, *SP* substance P, *SVF* stromal vascular fraction, *SWT* surgical waste tissue, *VEGF* vascular endothelial growth factor, *vWF* von Willebrand factor

### Bioengineering strategy

Unlike in vitro and in vivo vascularization strategies, which largely rely on cellular self-organization or the host environment, bioengineering approaches utilize advanced technologies to guide the formation of vascular structures in a more controlled manner. These methods enable improved spatiotemporal control, reproducibility, and scalability. In addition, they provide a versatile platform for regulating microenvironmental cues and modeling dynamic processes, thereby offering complementary advantages for investigating vascular function and disease-related pathophysiology.^94^

#### Organ-on-chip

Organ-on-chip represents an alternative platform for modeling vascularized bone. As a microphysiological system, it integrates microfluidics with 3D culture to reconstruct bone–vascular interactions under controlled conditions. This approach enables dynamic perfusion through microchannels, allowing precise and dynamic regulation of mass transport and microenvironmental cues.^95,96^

Vascularization in bone-on-chip systems is primarily achieved through two design strategies: patterned assembly and templating.^97^ Patterned assembly refers to the self-assembly of ECs into microvascular networks within a hydrogel matrix. For example, in bone-on-chip systems, incorporation of hydroxyapatite to mimic the bone microenvironment has been shown to promote endothelial sprouting within the matrix, leading to the formation of interconnected vascular networks.^98^ Compared with patterned assembly, which relies on biological self-organization and recapitulates emergent vascular behaviors, templating enables the generation of predefined vascular architectures and provides greater structural control. This is achieved by generating predefined microchannels with sacrificial templates, which are subsequently endothelialized to form perfusable lumen structures within bone matrices. For example, in a GC-induced bone-on-chip model, a cylindrical microchannel was created by inserting and removing a needle within an osteoblast-laden matrix, followed by EC seeding to form a continuous, lumenized microvessel with well-defined geometry (Fig. 3c).^14^

Biomaterials that support vascularization in bone-on-chips are often incorporated as hydrogels, which promote endothelial sprouting and microvascular network formation.^99^ General pro-angiogenic matrices, such as fibrin, collagen, and fibronectin, primarily support endothelial adhesion, migration, and network formation.^98–101^ Bone-specific materials, including hydroxyapatite, calcium phosphate, and decellularized bone matrix, also enhance vascularization and promote angiogenesis–osteogenesis coupling.^102–104^ Collectively, these materials enable bone-on-chip systems to better approximate the biochemical and mineral cues of the bone microenvironment.

Beyond fabrication strategies and biomaterial selection, microenvironmental cues constitute another important regulatory layer in the shaping of vascularization.^97^ Perfusion is widely recognized as a central feature of bone-on-chip platforms and is essential for establishing hemodynamic conditions.^101,102,105^ Shear stress activates ECs and supports angiogenic sprouting, while enhanced transport ensures nutrient delivery and waste removal.^106^ Flow also establishes biochemical gradients that guide vascular patterning, ultimately supporting the formation of interconnected, perfusable vascular networks.^107^ Oxygen can also be modulated within these systems. For example, Bersini et al. varied the chip thickness to establish spatial oxygen gradients, resulting in spatial differences in vascular networks.^100^ In addition, fibroblasts are frequently incorporated as stromal support cells, where they secrete pro-angiogenic growth factors and matrix proteins that promote endothelial sprouting and stabilize vascular networks.^98,108,109^ Neural components can also be integrated, where neuron-derived signals modulate vascular growth.^110^ Together, these microenvironmental factors provide dynamic, multilayered regulation, highlighting the unique advantage of bone-on-chip platforms for modeling vascularized bone.

Recently, vascularized bone-on-chips have evolved from simplified physiological models^98,100^ to systems capable of modeling bone–vascular pathophysiology,^102,110^ largely owing to the precise spatiotemporal control of perfusion, mass transport, and microenvironmental cues afforded by microfluidic systems.^95,96^ This has expanded their application in mechanistic studies of bone–vascular pathologies. Ji et al. developed a vascularized bone-on-chip that mimics the pre-metastatic niche, demonstrating that lung cancer cell invasion is associated with cortactin and Tks5 activation, leading to invadopodia formation and subsequent cytoskeletal remodeling that promotes invasive behavior.^103^ Smith et al. developed a vascularized osteochondral microphysiological system based on a biphasic bioreactor to model cartilage–bone crosstalk under inflammatory conditions.^111^ Despite these advances, most platforms, while capable of achieving basic perfusion and supporting cell transport, still fall short of recapitulating authentic blood flow function, particularly in incorporating circulating blood cellular components and oxygen transport capacity, as well as the full complexity of dynamic biochemical exchanges.^112^ Overall, bone-on-chip platforms represent a complementary approach to other vascularization strategies, offering superior control over perfusion and microenvironmental parameters, but still requiring further development to fully recapitulate the complexity of bone vasculature. To further summarize recent developments in this area, vascularized bone-on-chip models are compiled in Table 3.

**Table 3:** Summary of vascularized bone-on-a-chip models

Bone model	Vascular fabrication	Cell types	Biomaterials	Microenvironmental cues	References
Vascularized bone model	Patterned assembly	HUVEC; lung fibroblast	Fibrin; HA	Perfusion flow; fibroblast-mediated vascular support	^98^
Vascularized bone model	Patterned assembly	HUVEC; MSC	Fibrin; collagen I	Oxygen tension regulation	^100^
Breast cancer bone metastasis model	Patterned assembly	EC; BMSC; MDA-MB-231 breast cancer cell	Decellularized bone matrix	Perfusion flow; oxygen tension regulation; tumor microenvironment	^102^
Breast cancer bone extravasation model	Patterned assembly	HUVEC; BMSC; MDA-MB-231 breast cancer cell	Fibrin	Perfusion flow; tumor microenvironment	^105^
Bone tumor microenvironment angiogenesis model	Patterned assembly	HUVEC; fibroblast; SW620 colon cancer cell; MKN74 gastric cancer cell	Fibrin; HA	Tumor microenvironment	^242^
Vascularized bone model	Templating	HUVEC; osteoblast	GelMA; PEGDA; PEGDMA; SPELA; agarose	Perfusion flow	^101^
Inflammatory neurovascular bone model	Templating	HUVEC; DRG neuron; osteoclast	Collagen I; fibrin; laminin	IL-1β-mediated inflammation; neural regulation	^110^
Fracture healing model	Patterned assembly	MSC	Gelatin; USPION; poly-L-lysine	Perfusion flow; magnetic actuation	^243^
Osteoarthritis osteochondral interface model	Patterned assembly	BMSC; HUVEC; macrophage	GelMA; MeHA; gelatin; HA; fibrin	Perfusion flow; IL-1β/IL-6/TNF-α-mediated inflammation	^111^
Glucocorticoid-induced bone microvascular dysfunction model	Templating	Osteoblast; EC	Collagen I; fibrinogen; fibronectin; poly-L-lysine	Perfusion flow; glucocorticoid induction	^14^
Periodontitis bone–vascular interface model	Templating	BMSC; HUVEC; THP-1 monocyte	Fibronectin	Perfusion flow; LPS/TNF-α-mediated inflammation	^244^
Breast cancer bone metastasis model	Patterned assembly	HUVEC; BMSC; fibroblast; MDA-MB-231 breast cancer cell; neutrophil	Fibrin	Perfusion flow; tumor microenvironment	^109^
Osteoporosis-associated breast cancer bone metastasis model	Templating	Osteocyte; osteoblast; osteoclast; HUVEC; MDA-MB-231 breast cancer cell	Fibrin; fibronectin	Perfusion flow; osteoporotic condition	^245^
Osteoarthritis osteochondral interface model	Patterned assembly	Articular chondrocyte; BMSC; HUVEC	PEG hydrogel	IL-1β-mediated inflammation	^246^
Lung cancer bone metastasis model	Templating	MSC; osteoclast; HUVEC; A549 lung cancer cell	GelMA; HA; poly-D-lysine	Perfusion flow; tumor microenvironment	^103^
Osteoarthritis osteochondral interface model	Patterned assembly	Articular chondrocyte; osteoclast; EC; BMSC	Fibrin; calcium phosphate	IL-1β-mediated inflammation	^104^
Breast cancer bone metastasis model	Patterned assembly	HUVEC; MSC; lung fibroblast; MDA-MB-231 breast cancer cell; HL-60 cell	Fibrin; collagen I	Perfusion flow; fibroblast-mediated vascular support; tumor microenvironment	^108^
Glucocorticoid-induced bone microvascular dysfunction model	Patterned assembly	BMEC; lung fibroblast	Collagen I; HA	Perfusion flow; glucocorticoid induction; fibroblast-mediated vascular support	^15^
Endochondral ossification model	Patterned assembly	HUVEC; embryonic stem cell-derived sclerotome cell	Fibrin; collagen I	Perfusion flow	^76^
Lung cancer bone metastasis model	Patterned assembly	HUVEC; BMSC; THP-1 monocyte; A549 lung cancer cell	Fibrin	Perfusion flow; tumor microenvironment	^247^

*BMEC* bone microvascular endothelial cell, *BMSC* bone mesenchymal stromal cell, *DRG* dorsal root ganglion, *EC* endothelial cell, *GelMA* gelatin methacryloyl, *HA* hydroxyapatite, *HL-60* human promyelocytic leukemia cell, *HUVEC* human umbilical vein endothelial cell, *MeHA* methacrylated hyaluronic acid, *MSC* mesenchymal stromal cell, *PEG* poly(ethylene glycol), *PEGDA* poly(ethylene glycol) diacrylate, *PEGDMA* poly(ethylene glycol) dimethacrylate, *SPELA* star poly(ethylene glycol-co-lactide) acrylate, *THP-1* human monocytic leukemia cell line, *USPIO* ultrasmall superparamagnetic iron oxide

#### 3D bioprinting

3D bioprinting vascular strategies combine 3D printing, tissue engineering, and developmental biology to construct biomimetic vascularized tissues. This technique organizes cells and matrix materials into bioinks, which are then precisely printed under computer guidance to form predefined 3D vascular networks.^113^ Kerouredan et al. precisely printed high-density ECs onto hydrogels containing BMSCs, generating microvascular networks with defined architectures (Fig. 3d).^114^ Anada et al. used digital light processing to fabricate dual-hydrogel constructs, consisting of an outer ring of GelMA embedded with octacalcium phosphate and an inner ring containing HUVECs. This structure effectively promoted osteogenic differentiation of BMSCs and induced vascular network formation.^115^ Specifically, incorporating osteoinductive and angiogenic factors into bioinks further enhances osteogenic differentiation and vascularization. For example, bioinks containing VEGF-releasing microspheres promoted early angiogenesis and maintained long-term vascular stability.^116^

This strategy enables the fabrication of complex, customizable vascular branches, overcoming the uncontrolled variability of the vascular network in traditional co-culture methods.^117^ Moreover, preconstructing deep vascular networks can alleviate the limitations of nutrient diffusion within organoids. However, this technique requires precise control of printing parameters and careful optimization of bioink compositions, while the associated equipment requirements and technical complexity remain high.^118^

Despite rapid advances in these vascularization strategies, the quantitative evaluation of vascularization remains difficult to compare directly across studies. Metrics such as the number of branches and nodes, as well as total vessel length, are commonly reported; however, differences in imaging areas and magnifications limit cross-study comparability. These limitations highlight the need for more standardized and comparable metrics in future studies.

Collectively, these vascularization strategies provide the basis for the development of VBOs, which are increasingly being used for ONFH modeling and therapeutic exploration.

## VBOs for mechanistic investigation of ONFH

### Why VBOs represent a promising platform for studying ONFH

The pathogenesis of ONFH is multifactorial, including impaired bone remodeling, chronic inflammation, dysregulated lipid metabolism, and bone marrow edema, all intrinsically linked to vascular dysfunction.^12,13^ However, the complex pathophysiology of ONFH remains incompletely understood, and conventional experimental models fail to fully recapitulate it.^6^ Given the central role of vascular dysfunction in ONFH, which differs from other skeletal disorders, and the limitations of current experimental models, VBOs offer an emerging biomimetic model for the mechanistic investigation of ONFH.

#### Pathogenesis of ONFH: vascular injury as the central mechanism

The femur, the largest and one of the most highly vascularized bones in the human body, relies on a well-maintained vascular network to sustain its mechanical strength, metabolic activity, and regenerative capacity.^119^ Disruption of this vascular supply can therefore have profound consequences for bone viability. ONFH is a severe ischemic bone disorder with a high risk of disability.^1^ Various risk factors, including prolonged GC use, excessive alcohol consumption, and trauma, are known to impair vascular integrity and reduce blood supply to the femoral head, ultimately leading to ischemic bone injury and osteonecrosis.^120,121^

Risk factors and their associated early vascular injury are considered initiating events in ONFH, triggering multistep pathological cascades, including impaired bone remodeling, chronic inflammation, and dysregulated lipid metabolism.^12,122^ Osteocytes are embedded within the mineralized bone matrix and are highly sensitive to oxygen deprivation. Ischemia and hypoxia can directly impair osteocyte viability and disrupt bone remodeling, which further disturbs angiogenesis–osteogenesis coupling within the femoral head.^123,124^ Chronic inflammation is a hallmark of ONFH. Excessive production of inflammatory mediators and reactive oxygen species not only damages ECs and aggravates coagulation abnormalities but also exacerbates bone degeneration.^125,126^ In addition, dysregulated lipid metabolism, manifested by adipocyte hypertrophy and hyperplasia as well as hyperlipidemia, leads to perivascular lipid deposition.^127,128^ Collectively, impaired bone remodeling, chronic inflammation, and dysregulated lipid metabolism compromise vascular function and aggravate microcirculatory impairment, thereby promoting bone marrow edema, a key event in the progression of ONFH.^129^ Local microcirculatory impairment can obstruct venous outflow, leading to fluid accumulation within the confined marrow cavity and the development of bone marrow edema.^1,130^ As intraosseous pressure increases as a result, microvascular perfusion becomes further restricted. Sustained elevation of intraosseous pressure also exacerbates osteocyte death and promotes trabecular microfractures.^129^ Finally, these pathological cascades progressively disrupt the angiogenic–osteogenic coupling within the femoral head and, through a self-amplifying process, ultimately lead to structural collapse of the femoral head.^131^

Recent studies have revealed that lymphatic and blood vessels within bone form an integrated vascular network that cooperates in maintaining bone homeostasis and regulating inflammation. Dysfunction of the lymphatic system can further aggravate microcirculatory impairment in ONFH, thereby compromising tissue repair.^132,133^

In summary, the pathogenesis of ONFH is multifactorial, including impaired bone remodeling, chronic inflammation, dysregulated lipid metabolism, bone marrow edema, and impaired lymphatic drainage, all of which are intrinsically linked to vascular dysfunction. Despite the recognized central role of vascular injury in ONFH,^134–137^ the precise mechanisms underlying vascular impairment remain incompletely understood. The vascular-centered pathogenesis highlights the specific suitability of VBOs for studying ONFH.

#### Limitations of conventional models and the potential of VBOs

Conventional experimental models of ONFH mainly include animal models and cell culture models. Animal models are typically established in rodents or rabbits through pharmacological induction (most commonly GCs or alcohol) or surgical interruption of the blood supply. In vitro models commonly rely on cultured cells.^138^ These models have provided valuable insights into the pathogenesis of ONFH. However, these models fail to capture the human pathophysiology of ONFH fully. Animal models are limited by interspecies differences between animals and humans, including endocrine regulation, metabolic processes, and hormone receptor expression. Moreover, differences in bone architecture and vascular supply further reduce their translational relevance.^139^ They are also inherently limited in recapitulating key pathological features such as bone marrow edema and femoral head collapse.^6^ Moreover, conventional cell culture models lack the three-dimensional architecture and cannot reproduce the complex inter-tissue interactions.

**Fig. 4:**
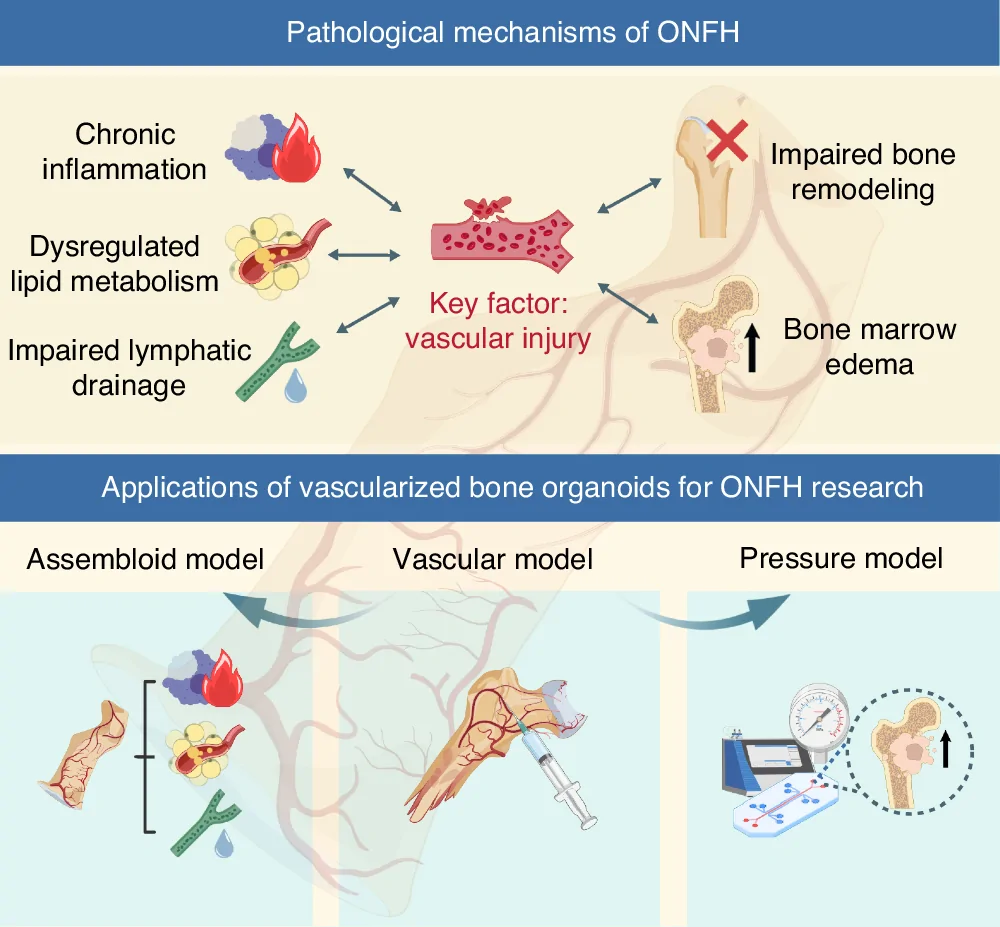
Pathological mechanisms of ONFH and applications of VBOs in mechanistic studies of ONFH. Vascular injury represents the initiating and central pathological event in ONFH. The disease process also involves impaired bone remodeling, chronic inflammation, dysregulated lipid metabolism, bone marrow edema, and impaired lymphatic drainage, all closely linked to vascular damage. VBOs provide a biomimetic platform to dissect ONFH pathogenesis. Vascular models enable investigation of disrupted angiogenesis–osteogenesis coupling, assembloid models support exploration of interactions with inflammation, lipid metabolism, and lymphatic drainage, and pressure models help elucidate mechanisms of intraosseous pressure dynamics associated with bone marrow edema

Consequently, these models remain insufficient for investigating the complex vascular-centered mechanisms underlying ONFH pathogenesis in humans.^6,138^ Fully recapitulating the complexity of ONFH requires consideration of disrupted angiogenesis–osteogenesis coupling, vascular-centered pathological cascades, and alterations in intraosseous pressure. More recently, VBOs, which recapitulate key features of the human vascularized bone, have emerged as a promising strategy for elucidating the pathological mechanisms of ONFH. Specifically, basic VBO models that integrate skeletal and vascular components enable investigation of disrupted angiogenesis–osteogenesis coupling.^140^ Furthermore, with the integration of additional tissue components, VBO-based assembloids facilitate the study of vascular -centered pathological cascades.^141^ In addition, with precise biomechanical monitoring and modulation, microfluidic VBO systems enable the investigation of intraosseous pressure dynamics associated with bone marrow edema. This provides insights into the mechanisms underlying bone marrow edema in ONFH progression.^142^ Collectively, these capabilities position VBOs as a promising platform for advancing the mechanistic understanding of ONFH (Fig. 4).

Accordingly, the application and ongoing exploration of VBO-based models in investigating the pathological mechanisms of ONFH are discussed in detail below. Given that steroid-induced ONFH (SONFH) is the most common and extensively studied subtype, SONFH is used herein as a representative model.

### Angiogenesis–osteogenesis coupling in VBO models

The femoral head is highly vascularized, with H-type vessels playing a critical role in the coupling of angiogenesis and osteogenesis.^143,144^ GC inhibits the formation of these vessels by downregulating angiogenic regulators such as HIF-1α, PDGF-BB, and VEGF, thus disrupting the coupling between angiogenesis and osteogenesis and diminishing the femoral head’s regenerative capacity.^123^ Hormone-induced VBOs provide an emerging platform for elucidating how GC disrupts the coupling between angiogenesis and osteogenesis. Using VBOs, Lee et al. demonstrated that GC impairs osteogenesis and bone microvascular barrier function by interfering with endothelial-osteoblast interactions. These GC-induced alterations are mediated by activation of the MAPK signaling cascade (ERK/p38/JNK), which induces phosphorylation of connexin43 at Ser282.^14^ Fan et al. showed that hormone-induced vascularized bone chips exhibited elevated TNF-α expression in necrotic regions of the femoral head, consistent with clinical observations. Furthermore, the truncated APT19 aptamer mitigated TNF-α-mediated apoptosis in bone microvascular ECs and attenuated associated cytoskeletal disorganization.^15^ These models recapitulate the key pathological features of hormone-induced vascular dysfunction and osteonecrosis, enabling detailed investigations of GC–vascular–bone interactions.^8,145^

Different vascularization strategies exhibit distinct features for investigating GC-induced disruption of angiogenesis–osteogenesis coupling. Co-culture systems that directly reconstitute vascular–bone interactions serve as fundamental models for mechanistic studies.^94^ During the repair of ONFH, fibrovascular invasion is accompanied by endochondral bone formation.^146^ Accordingly, endochondral ossification strategies help elucidate the mechanisms underlying the ineffective replacement of necrotic bone by newly formed bone during ONFH repair. Microfluidic chips enable programmable GC delivery that reproduces daily hormone fluctuations under different dosing regimens, allowing investigation of GC exposure-dependent effects.^147^ Moreover, given the highly vascularized nature of the femoral head, scaffold-assisted strategies and in vivo vascularization facilitate the dense vascular networks that better recapitulate the rich vascularization of the femoral head.

### Multi-tissue crosstalk in VBO-based assembloids

The pathogenesis of SONFH involves immune dysfunction, dysregulated lipid metabolism, and impaired lymphatic drainage, ultimately leading to microcirculatory dysfunction and osteonecrosis.^13,148^ Recognizing the limitations of single-organoid systems recapitulating complex inter-tissue crosstalk, researchers have developed organoid assembloids-integrated 3D constructs composed of multiple organoid types or cell lineages that enable inter-tissue interactions.^141^ The concept was first proposed by Birey et al., who generated a dorsal–ventral forebrain assembloid that facilitated neuronal connectivity between glutamatergic and GABAergic populations, representing a significant advance in modeling tissue–tissue communication.^149^ This section focuses on immune dysfunction, dysregulated lipid metabolism, and impaired lymphatic drainage, highlighting the potential of vascularized bone assembloid models for elucidating the pathogenesis of SONFH.

#### Immune dysfunction

In the chronic inflammatory phase of SONFH, sustained M1 macrophage activation drives inflammation and hinders tissue repair by releasing proinflammatory cytokines such as TNF-α, IL-6, and IL-1β.^150,151^ This process is triggered by damage-associated molecular patterns released from necrotic bone and vascular endothelium, which activate a downstream inflammatory cascade, particularly the TLR4/NF-κB signaling axis.^125^ To date, most immunological studies of SONFH have focused on macrophages, particularly their polarization and inflammatory regulation. However, emerging evidence suggests that additional immune cell types also participate in disease progression. Excessive neutrophil extracellular traps have been observed in necrotic regions, disrupting endothelial integrity by inducing endothelial–mesenchymal transition.^152–154^ Dendritic cell infiltration appears diminished in SONFH,^155^ and IFN-λ1 secreted by dendritic cells attenuates osteoclastogenesis through NF-κB inhibition, JAK–STAT activation, and NLRP3 inflammasome suppression.^156^ Together, these findings underscore that SONFH pathogenesis involves a coordinated immune network. Co-culture models offer a powerful platform to systematically dissect the roles of diverse immune cell types in SONFH.

Immune cell–VBO assembloids have been utilized to elucidate the inflammatory mechanisms underlying various bone disorders. For instance, in a study of breast cancer bone metastasis, VBOs co-cultured with neutrophils showed that metastatic tumor cells facilitated the extravasation of neutrophils with an antitumor phenotype, thereby suppressing the destruction of vascularized bone structures.^109^ In another study, Neto et al. developed a neurovascular bone assembloid incorporating inflammatory osteoclasts. They showed that these osteoclasts secreted pro-angiogenic factors, increasing vascular permeability and promoting aberrant neuronal axon extension into vascular regions.^110^ Collectively, immune cell–VBO assembloids enable direct investigation of the immune–bone–vascular interactions underlying SONFH pathogenesis.^157^

#### Dysregulated lipid metabolism

GC disrupts the balance between osteogenic and adipogenic differentiation of BMSCs.^158^ Hypertrophic and proliferative adipocytes secrete various adipokines and extracellular vesicles that promote the development of SONFH.^159,160^ For example, PAI-1 impairs fibrinolysis by binding to tPA, leading to hypercoagulability,^161^ and microvesicles enriched in *miR-148a* suppress the Wnt5a/Ror2 signaling pathway to inhibit osteogenesis.^162^ Other paracrine signaling mechanisms implicated in SONFH remain to be elucidated. Moreover, Serum lipid levels are elevated in patients with SONFH, including cholesteryl esters, phosphatidylethanolamines, glycerophospholipids, and other lipid species.^163–165^ Hyperlipidemia, as an important contributor to vascular dysfunction, is known to promote atherosclerosis through multiple mechanisms, including endothelial dysfunction, vascular inflammation, and oxidative stress.^166,167^ Nevertheless, the specific mechanisms linking GC-induced hyperlipidemia to osteonecrosis remain unclear.^168^

Yang et al. established a liver–adipose assembloid, in which medium circulation enabled bidirectional molecular crosstalk (such as lipids and exosomes) between the two organoid types. This assembloid demonstrated that adipose tissue-derived extracellular vesicles promoted albumin secretion in liver organoids.^169^ Similarly, another study found that adipocyte inflammation induces hepatic insulin resistance via the TNF-α/NF-κB signaling axis.^170^ Furthermore, upon exposing brain and lung organoids to bioactive lipids such as lysophosphatidic acid, researchers identified a lipid-mediated mechanism that regulates apicobasal polarization in tissues.^171^ Extending these approaches to SONFH research by co-culturing adipose tissue with VBOs or by exposing VBOs to SONFH-associated lipid metabolites may more precisely model the pathological lipid microenvironment affecting bone vascular units. Such models could help elucidate the specific paracrine and metabolic pathways that drive SONFH.

#### Impaired lymphatic drainage

Recent studies have confirmed the presence of lymphatic vessels within bone tissue.^133^ These vessels form an integrated network with blood vessels that support bone homeostasis, modulate inflammation, and facilitate tissue repair.^132,172^ Although microcirculatory disturbance is a major pathological feature of SONFH,^12^ the role of lymphatic drainage in this process remains poorly understood. It remains uncertain whether GC directly or indirectly influences intraosseous lymphatic vessels, further exacerbating microcirculatory impairment.

Similar lymphatic-related assembloids have been developed in other fields. Sato et al. discovered that snake venom markedly enhances the permeability between lymphatic and blood vessels within the lymphatic-vascular assembloid, providing a rapid approach for evaluating antivenom efficacy.^173^ Wang et al. constructed a leiomyoma assembloid containing lymphatic ECs to model lymphangioleiomyomatosis, demonstrating that myoma cells recruit lymphatic ECs and promote lymphatic tube formation via the VEGF-D/VEGFR3 signaling pathway.^174^ Given that lymphatic drainage is an essential component of the microcirculatory system,^132^ incorporating lymphatic vessels into VBOs could enable the reconstruction of a more complete microcirculatory model and help elucidate whether lymphatic dysfunction and vascular injury act synergistically to aggravate SONFH.

Collectively, VBO-based assembloids extend basic VBO models by incorporating additional tissue components, thereby enabling investigation of multi-tissue crosstalk in ONFH. Accordingly, the distinct features of the vascularization strategies for disease modeling discussed in Section 3.2 are also applicable here. In addition, scaffold-assisted strategies enable incorporation of materials that modulate pathological cascades, such as those promoting adipogenic differentiation or M1 macrophage polarization.^175,176^ Moreover, microfluidic chips are widely used in assembloid systems because they enable the integration of multiple cell types and support their crosstalk through controlled medium circulation.^177,178^

### Intraosseous pressure in microfluidic VBO systems

In the early stage of SONFH, bone marrow edema and elevated intraosseous pressure are commonly observed.^179^ Excessively elevated intraosseous pressure impairs microcirculatory perfusion, whereas abnormally low pressure impedes bone tissue repair.^180^ However, the specific influence of dynamic changes in intraosseous pressure on SONFH development remains poorly understood.^129^ To date, only one study has demonstrated that early elevation of intraosseous pressure upregulates Piezo1 expression in BMSCs, leading to enhanced intracellular calcium influx and subsequently stimulating osteogenesis and angiogenesis through the CAM–NFAT1 signaling pathway.^130^ Furthermore, the mechanisms underlying bone marrow edema and elevation of intraosseous pressure remain largely unclear. Vascular disruption and marrow adipocyte accumulation are considered key contributors, but additional studies are required to clarify how GC contributes to bone marrow edema and increased intraosseous pressure.^130^

Bone marrow edema is a key pathological manifestation of ONFH and is closely associated with increased intraosseous pressure. This pressure reflects fluid accumulation within the bone marrow cavity and provides a quantifiable, controllable parameter for modeling edema conditions. However, these features remain insufficiently captured across existing experimental models. This is partly due to the difficulty of reproducing confined fluid accumulation and pressure dynamics within the bone marrow cavity.^181^ Microfluidic systems may offer a potential strategy, as they enable precise control of fluid flow and pressure. Studies in other tissues have demonstrated the feasibility of pressure modulation and real-time monitoring within tissues, suggesting that similar approaches could be adapted to model marrow pressure and edema in marrow-on-chip platforms.^142,182,183^ For instance, precise pressure modulation and real-time monitoring within 30–200 mmHg have been achieved in retinal tissue by pressurizing a porous agarose matrix within a microfluidic platform with sensor-based monitoring.^184^ However, current marrow-on-a-chip platforms have not yet incorporated microfluidic pressure systems. Incorporating microfluidic pressure systems into SONFH research may enable more physiologically relevant simulation of the mechanical and fluidic milieu within bone, facilitating investigation of how dynamic marrow pressure conditions influence bone cell activity, vascular function, and bone regeneration. Ultimately, such approaches may help to elucidate the mechanisms linking GC-induced intramedullary pressure abnormalities to the initiation and repair of osteonecrosis.

## Applications of VBOs in the treatment of ONFH

### Evaluating pro-angiogenic therapies

Vascular injury constitutes the initiating and predominant mechanism underlying ONFH.^134,135^ Recent studies have underscored the therapeutic potential of targeting endothelial dysfunction, modulating angiogenic signaling, and correcting coagulation disorders in the management of ONFH.^12,122^ Multiple therapeutic strategies—including pharmacological intervention, genetically modified stem cell therapy, physical stimulation, and scaffold-based implantation—have been developed to regulate vascular-associated pathways.^185–189^ For instance, hydrogels releasing angiogenic peptides^190^ and the transplantation of *HIF-1α*-modified BMSCs^145^ have been shown to promote angiogenesis and attenuate osteonecrotic progression.

Although numerous vascular-targeting strategies have been explored in preclinical studies, their clinical translation remains hampered by the inherent limitations of conventional animal models.^122^ The VBO model provides a human-relevant platform for evaluating vascular-targeted therapies. Li et al. established a vascularized organoid derived from tissue obtained during total hip arthroplasty, in which icariin effectively inhibited dexamethasone-induced apoptosis of bone microvascular ECs. This effect was associated with reduced activation of cleaved caspase-3/7.^16^ Fan et al. also developed a hormone-induced vascularized bone chip to model ONFH, into which the truncated APT19 aptamer targeting TNF-α was incorporated to evaluate its therapeutic efficacy. Treatment with the aptamer significantly suppressed endothelial apoptosis, restored cytoskeletal integrity, and promoted angiogenesis.^15^ By directly applying pro-angiogenic strategies to VBO-based models, researchers can simultaneously evaluate their therapeutic efficacy and biosafety. Notably, these models can be further miniaturized into high-throughput platforms, enabling systematic screening of pro-angiogenic therapies.^191^ In summary, pro-angiogenic therapy holds great potential for ONFH treatment, and VBO-based models provide an efficient and translationally relevant platform for target validation and therapeutic optimization.

### Regenerative medicine

The current standard of care for early ONFH typically includes core decompression, pharmacological interventions, and physical therapy.^192^ Despite partial symptomatic relief from early interventions, these strategies remain insufficient to restore functional perfusion and promote structural regeneration, frequently culminating in femoral head collapse and eventual joint replacement.^193,194^ To enhance regeneration, autologous osteoblastic cell implantation has been investigated as a therapeutic approach.^195^ However, this strategy has not shown superiority over standard care, suggesting that osteoblastic cell delivery alone may be inadequate to overcome the ischemic microenvironment of ONFH.^196,197^ This suggests that VBOs may serve as promising implantable constructs with the potential to support vascular regeneration and bone repair.

VBOs may have regenerative potential for ONFH by integrating vascular and osteogenic components to repair bone tissue. Following implantation, preformed vascular networks within VBOs may anastomose with host vasculature, potentially re-establishing perfusion in ischemic regions and thereby supporting coupled angiogenesis and osteogenesis.^198^ Beyond vascular restoration, VBOs may help optimize the local microenvironment by enhancing oxygen and nutrient delivery while facilitating the clearance of metabolic waste and inflammatory mediators. Consequently, these effects may help alleviate hypoxia and attenuate local inflammation, thereby establishing a microenvironment favorable for bone regeneration.^199^ Structurally, with appropriate biomechanical properties, VBOs may provide partial mechanical support, helping to preserve femoral head integrity and slow trabecular degeneration.^42^ Furthermore, their integrated vascular networks may support sustained tissue remodeling and bone homeostasis, although their long-term effects remain to be established.^45^

VBOs have been shown to markedly enhance vascularization and osteogenesis in mouse femoral defect models.^199^ However, the application of implantable VBOs for ONFH remains at an early stage, and several limitations should be considered. These include maintaining the viability and long-term functionality of large-scale VBOs, adapting implanted VBOs to the hostile ONFH microenvironment, and mitigating immune rejection.^200–202^

### Personalized treatment

Given the multifactorial nature of ONFH, genetic susceptibility interacts with local microenvironmental factors to shape angiogenic capacity, osteogenic potential, lipid metabolism, and immune responses, among other related pathogenic processes (summarized in Table 4). Substantial inter-individual heterogeneity, partly driven by genetic variability, exists in disease progression and therapeutic responsiveness among ONFH patients, underscoring the importance of developing genetically informed, patient-specific models for precision treatment.^203–205^

**Table 4:** Summary of genes and related mechanisms in ONFH

Genes	Related mechanisms	References
*TF, VEGF, IGFBP3, and ACE*	Angiogenesis and hypoxia	^248^
*C3435T* and *G2677T/A*	Steroid resistance-related variants	^249^
*SMAD3*	Osteogenic and adipogenic differentiation	^250^
*APOE*	Lipid metabolism	^251^
*PPARG, RUNX2* and *COL2A1*	Adipogenesis differentiation	^252^
*IL-1α, TGF-β, IL-10 and TNF-α*	Inflammation and osteocyte apoptosis	^253^
*TNFSF10, PTEN* and *BCL2L1*	Autophagy	^254^
*GYPA, TMCC2* and *BPGM*	Erythrocyte function and immune regulation	^255^
*NLRP1*	Pyroptosis	^256^
*NCF2* and *SLC2A1*	Ferroptosis	^257^
*RPL6, RPL9* and *RPL23*	Autophagy	^258^
*SLC2A1*	Apoptosis and osteoblast ossification	^259^
*STAT3, IL1B, TLR2* and *CASP1*	Endoplasmic reticulum stress	^260^
*BID, FTH1, LACTB, PDK3, RAB5IF, SOD2* and *SQOR*	Mitochondria regulation	^261^

Patient-derived iPSCs provide a powerful genetically informed tool for individualized disease modeling.^206,207^ Patient-derived iPSCs enable the establishment of VBOs that preserve the genetic background of the donor, thereby recapitulating individual differences in angiogenic capacity, osteogenic potential, and other related pathogenic processes. Such personalized VBOs enable the evaluation of differential therapeutic responses across distinct genetic backgrounds.

In addition, the integration of CRISPR Cas9 genome editing into organoid systems enables precise modeling of genetic alterations.^208,209^ By introducing pathogenic mutations, CRISPR enables the generation of the VBOs that capture gene-specific disease phenotypes.^210^ Importantly, these modified VBOs not only enable interrogation of susceptibility pathways underlying ONFH pathogenesis but also provide clinically relevant drug-response data, facilitating personalized drug screening and toxicity evaluation. For instance, by regulating the *PPARG/RUNX2* regulatory axis in VBOs through CRISPR-Cas9, this approach enables modeling of the osteogenic-adipogenic imbalance and the accompanying vascular dysfunction that contribute to ONFH. Selective activation of *PPARG* can promote adipogenic differentiation and recapitulate marrow fat accumulation, whereas upregulation of *RUNX2* promotes osteogenic differentiation and restores regenerative capacity. This genetically defined VBO enables the mechanistic dissection of ONFH under lipid metabolism-associated susceptibility and facilitates the identification of genetically informed, personalized therapeutic targets.

Moreover, VBOs can be miniaturized into scalable formats suitable for high-throughput CRISPR perturbation and small molecule screening.^211,212^ This scalability supports systematic discovery of susceptibility pathways and accelerates the development of personalized therapeutic strategies for ONFH.

## Summary and future perspectives

**Fig. 5:**
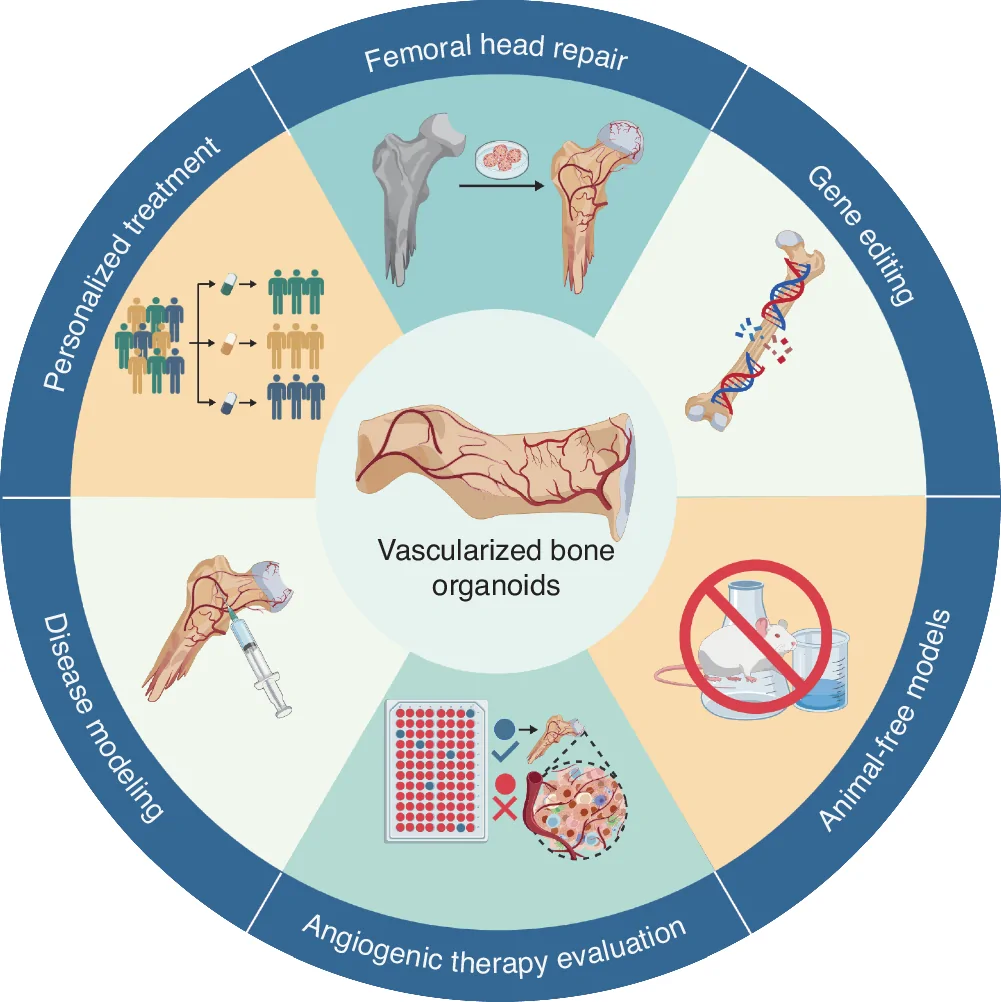
Summary of applications of VBOs in ONFH. VBOs provide versatile platforms for elucidating the pathogenesis of ONFH and developing novel therapeutic strategies. They hold promise for femoral head repair, gene editing, animal-free modeling, angiogenic therapy evaluation, disease modeling, and personalized treatment

In this review, we comprehensively delineate the vascularization strategies employed in bone organoids. The co-culture approach establishes basic angiogenesis–osteogenesis coupling but remains limited in reconstructing three-dimensional vascular networks. Given the highly mineralized nature of bone tissue, scaffold-assisted vascularization has emerged as the most widely adopted strategy. The endochondral ossification-based approach mimics the physiological process of bone development; however, the process is prolonged and technically challenging. In vivo vascularization strategies are often integrated with co-culture or scaffold-assisted methods to enhance vascular maturity and functionality. Organ-on-chip technology serves as a powerful microphysiological platform that enables dynamic simulation of vascular perfusion. Meanwhile, 3D bioprinting allows precise, customizable fabrication of complex branched vascular networks but is constrained by high technical thresholds, limiting its broader application in bone organoid models. Additionally, we summarize advances in vascularized biomaterials for bone tissue engineering, providing a reference framework for the future design of vascularized scaffolds for bone organoids. We also discussed the application of VBOs in ONFH, using SONFH as a representative example. Basic VBO models enable investigation of disrupted angiogenesis–osteogenesis coupling. By incorporating immune cells, adipocytes, or lymphatic components, assembloid models facilitate investigation of the synergistic pathological mechanisms of SONFH, including immune dysfunction, dysregulated lipid metabolism, and impaired lymphatic drainage. Microfluidics-mediated pressure models further enable mechanistic interrogation of intraosseous pressure dynamics associated with bone marrow edema. Importantly, while current studies primarily focus on SONFH, VBOs may also provide mechanistic insights into ONFH arising in other clinical contexts, including traumatic cases, alcohol-related cases, and systemic disorders such as sickle cell disease, SLE, GVHD, HIV infection, and radionecrosis, supporting their broader applicability across diverse etiologies. From a therapeutic perspective, VBOs provide a promising platform for preclinical evaluation of pro-angiogenic therapies. VBOs may have regenerative potential for ONFH—they may promote coupled angiogenesis and osteogenesis, optimize the local microenvironment, enhance bone stability, and maintain long-term efficacy. Furthermore, patient-derived organoids open avenues for personalized therapeutic development in the treatment of ONFH (Fig. 5). Despite ongoing challenges in clinical translation, VBOs hold substantial promise for advancing both mechanistic insight and therapeutic development in ONFH.

**Fig. 6:**
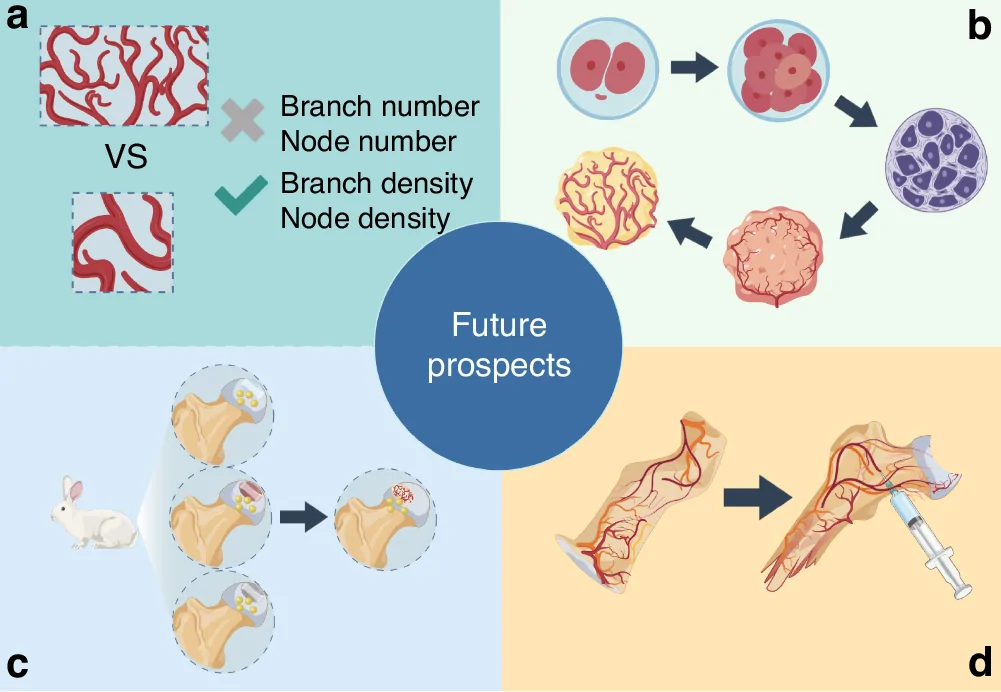
Future prospects for VBOs and their applications in ONFH. **a** Establishing standardized vascular parameters, such as branch and node density, is essential for improving the comparability of bone organoid vascularization studies. **b** VBOs can be engineered by recapitulating the spatiotemporal signaling dynamics of embryonic development, thereby enabling the generation of bone-specific vascular phenotypes. **c** In contrast to conventional scaffolds, VBO-based scaffolds recapitulate the microarchitecture of native bone tissue, offering superior physiological relevance and translational potential for vascular repair and functional regeneration of the femoral head. **d** Neural-vascularized bone organoids not only more comprehensively mimic the microenvironment of bone development but also provide new insights into the neurovascular interactions underlying ONFH

As vascularization strategies for bone organoids continue to evolve, the development of standardized vascular assessment frameworks based on consensus will be critical. The establishment of standardized vascular metrics—such as branch and node density—will be essential for data harmonization to enable robust cross-study comparisons (Fig. 6a).

The vascularization of bone organoids still faces a fundamental challenge: the inability to reproduce bone-specific vascular phenotypes, such as H-type and L-type vessels.^86^ Recent studies published in *Cell* and *Science* have shown that reconstructing the spatiotemporal dynamics of embryonic signaling pathways can generate vascularized organoids in which parenchymal tissues and vascular networks arise from a common iPSC origin, with the vascular compartment developing organ-specific phenotypes.^213,214^ The resulting vascularized lung organoids recapitulate human fetal lung architecture, featuring alveolar capillary-like vessels capable of gas exchange similar to human pulmonary microvessels.^214^ Inspired by these advances, by sequentially modulating key developmental cues, such as BMP, WNT, and IHH signaling.^215,216^ iPSCs can be directed to generate VBOs containing skeletal, vascular, and immune lineages by recapitulating developmental processes of bone formation, with the vascular compartment exhibiting bone-specific phenotypes. Quantification of such vascularization should include in vitro assessment of specific vascular phenotypes and in vivo validation of bone-related vascular functions, particularly the enhanced support for vascular–osteogenic coupling relative to conventional vasculature (Fig. 6b).

In the field of bone tissue engineering, a variety of scaffolds—including bioceramics, hydrogels, and synthetic polymers—have been utilized to modulate the pathological microenvironment of ONFH by delivering therapeutic agents or growth factors.^217^ At present, the fabrication of bone organoids still relies on various biomaterials, which also serve as carriers for the controlled release of bioactive molecules. In contrast to conventional scaffolds, VBO-based scaffolds recapitulate the native bone microarchitecture and exhibit superior physiological relevance and translational potential in facilitating vascular repair and functional regeneration of the femoral head (Fig. 6c).

During bone growth and regeneration, nerve fibers invade the tissue before vascular ingrowth and actively orchestrate angiogenesis through neurogenic signals.^218^ Our group demonstrated that GC suppresses sympathetic neural activity, thereby inhibiting PFKFB3-driven glycolysis in ECs via the Adrb2/cAMP/CREB pathway, which ultimately induces vascular apoptosis and the loss of H-type vessels.^219^ Collectively, the neurovascular-osteogenic axis is pivotal for maintaining bone homeostasis.^220^ Building on this concept, we envision a next-generation model with enhanced biomimetic fidelity—neurovascularized bone organoids. By incorporating neural components into bone organoids, this system recapitulates the developmental cascade of neural guidance, subsequent vascularization, and concomitant osteogenesis. Such an advanced platform may more comprehensively mimic the microenvironment of bone development and provide mechanistic insight into neurovascular interplay in ONFH (Fig. 6d).
